# Laccases: structure, function, and potential application in water bioremediation

**DOI:** 10.1186/s12934-019-1248-0

**Published:** 2019-11-14

**Authors:** Leticia Arregui, Marcela Ayala, Ximena Gómez-Gil, Guadalupe Gutiérrez-Soto, Carlos Eduardo Hernández-Luna, Mayra Herrera de los Santos, Laura Levin, Arturo Rojo-Domínguez, Daniel Romero-Martínez, Mario C. N. Saparrat, Mauricio A. Trujillo-Roldán, Norma A. Valdez-Cruz

**Affiliations:** 1 Departamento de Ciencias Naturales, Universidad Autónoma Metropolitana, Unidad Cuajimalpa, Av. Vasco de Quiroga 4871, Col. Santa Fe Cuajimalpa, C.P. 05348 Mexico City, Mexico; 2 Departamento de Ingeniería Celular y Biocatálisis, Instituto de Biotecnología, Universidad Nacional Autónoma de México, Av. Universidad 2001 Chamilpa, 62210 Cuernavaca, Morelos Mexico; 3 Programa de Investigación de Producción de Biomoléculas, Departamento de Biología Molecular y Biotecnología, Instituto de Investigaciones Biomédicas, Universidad Nacional Autónoma de México, AP. 70228, Mexico City, CP. 04510 Mexico; 4 Facultad de Agronomía, Universidad Autónoma de Nuevo León, Francisco Villa, 66059 Colonia Ex hacienda El Canadá, General Escobedo, Nuevo León Mexico; 5 Laboratorio de Enzimología, Facultad de Ciencias Biológicas, Universidad Autónoma de Nuevo León, Pedro de Alba y Manuel L. Barragán, Cd. Universitaria, 66451 San Nicolás de los Garza, Nuevo León Mexico; 6 Laboratorio de Micología Experimental, DBBE, Facultad de Ciencias Exactas y Naturales, Universidad de Buenos Aires, INMIBO-CONICET, Ciudad Universitaria, Pabellón 2, Piso 4, C1428BGA Ciudad Autónoma de Buenos Aires, Argentina; 7 Instituto de Fisiología Vegetal (INFIVE), Universidad Nacional de La Plata (UNLP)-CCT-La Plata-Consejo Nacional de Investigaciones Científicas y técnicas (CONICET), Diag. 113 y 61, 327CC, 1900, La Plata, Argentina; 8 Instituto de Botánica Spegazzini, Facultad de Ciencias Naturales y Museo, Universidad Nacional de La Plata, 53 # 477, 1900, La Plata, Argentina

**Keywords:** Bioremediation, Water bodies, Laccases, Emerging contaminants

## Abstract

The global rise in urbanization and industrial activity has led to the production and incorporation of foreign contaminant molecules into ecosystems, distorting them and impacting human and animal health. Physical, chemical, and biological strategies have been adopted to eliminate these contaminants from water bodies under anthropogenic stress. Biotechnological processes involving microorganisms and enzymes have been used for this purpose; specifically, laccases, which are broad spectrum biocatalysts, have been used to degrade several compounds, such as those that can be found in the effluents from industries and hospitals. Laccases have shown high potential in the biotransformation of diverse pollutants using crude enzyme extracts or free enzymes. However, their application in bioremediation and water treatment at a large scale is limited by the complex composition and high salt concentration and pH values of contaminated media that affect protein stability, recovery and recycling. These issues are also associated with operational problems and the necessity of large-scale production of laccase. Hence, more knowledge on the molecular characteristics of water bodies is required to identify and develop new laccases that can be used under complex conditions and to develop novel strategies and processes to achieve their efficient application in treating contaminated water. Recently, stability, efficiency, separation and reuse issues have been overcome by the immobilization of enzymes and development of novel biocatalytic materials. This review provides recent information on laccases from different sources, their structures and biochemical properties, mechanisms of action, and application in the bioremediation and biotransformation of contaminant molecules in water. Moreover, we discuss a series of improvements that have been attempted for better organic solvent tolerance, thermo-tolerance, and operational stability of laccases, as per process requirements.

## Introduction

**Fig. 1:**
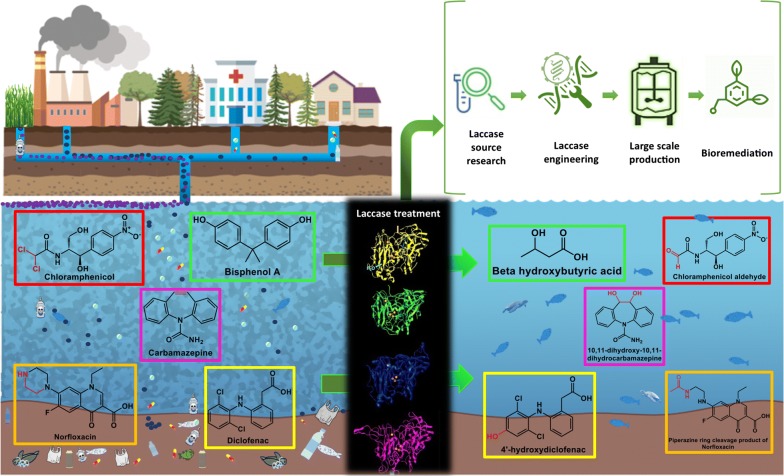
Schematic illustration of the potential sources of water contaminants and their bioremediation by laccases. Emerging contaminants such as antibiotics, endocrine disruptors, dye-based pollutants and pharmaceutical drugs are often released into the environment causing harmful impacts and health problems to humans and other animals, water treatment with laccases and their biotechnological approaches generate less-toxic, inert or fully degraded compounds

Urbanization and industrialization have resulted in a serious contamination of water bodies, causing harmful effects to ecosystems. Biotechnologists around the world are researching and developing innovative tools and non-polluting processes to correct the effect of global pollution. However, this is challenging, owing to the quantity and diversity of pollutant molecules discharged into water bodies [1], such as plastics, herbicides, fertilizers, synthetic dyes, polycyclic aromatic hydrocarbons (PAHs), chlorinated paraffin phthalates, and others, such as the so-called emerging pollutants, which may include pharmaceuticals (i.e. pain relievers, antibiotics, hormones, endocrine disruptors), plasticizers, and compounds contained in self-care products, among others [2–8]. Different treatment approaches have been explored, ranging from physical and chemical methods to biotechnological strategies (such as the use of laccase enzymes), to retain or transform these molecules into less harmful ones [9, 10]. The pollution of water bodies is a technical, social, and environmental challenge, attributable to continuous population increase and limited waste elimination strategies coupled with poor public management of water contaminants [4, 5, 11]. In fact, a variety of molecules originating from home and industry are released into water bodies without regulation; including emerging pollutants suspected to have effects on the environment and health (Fig. 1) [1, 12–14].

**Fig. 2:**
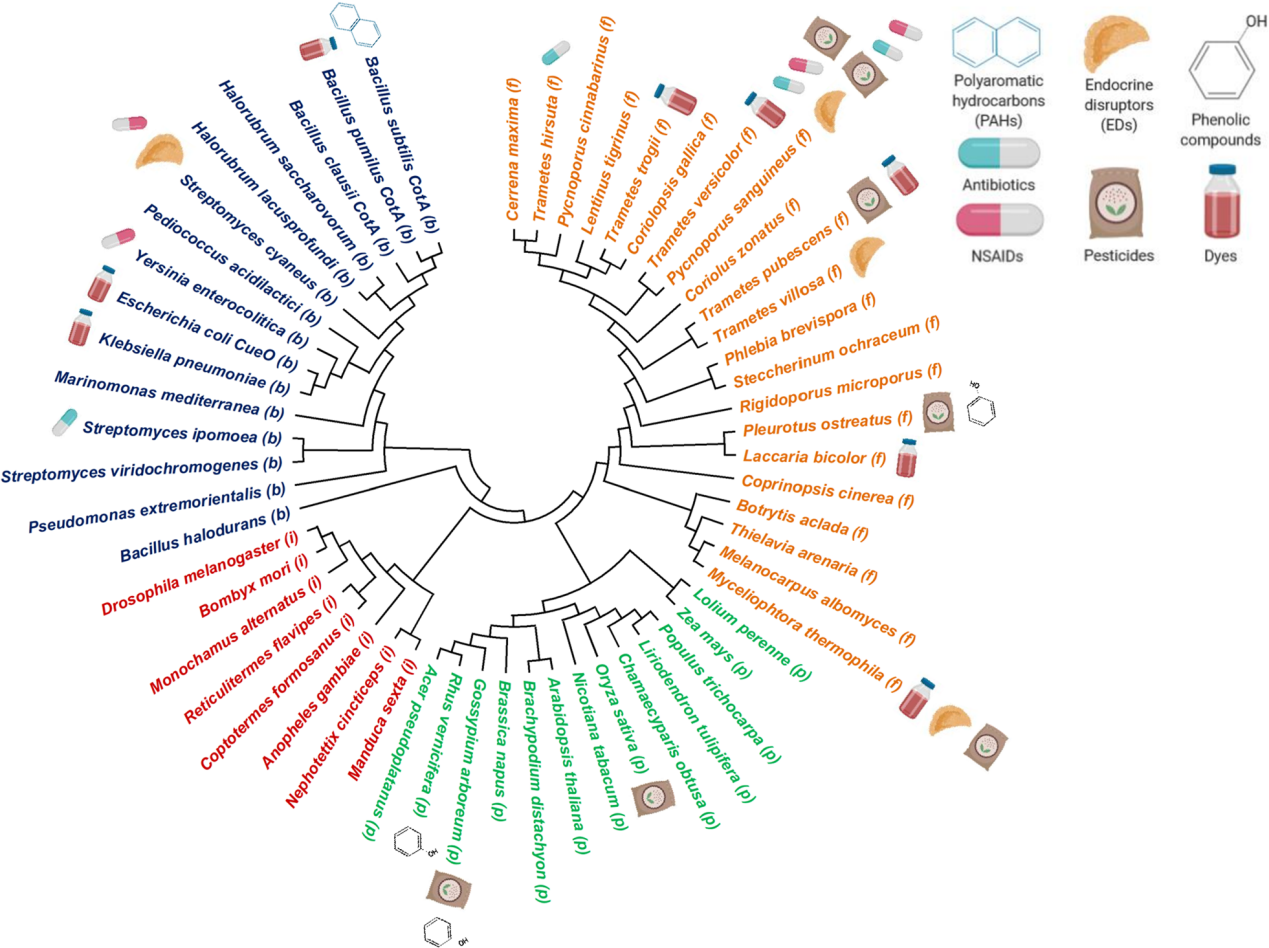
Phylogenetic tree constructed with some of the different organism sources of laccases, as well as some of their applications in bioremediation. According to their bacterial, insect, plant or fungal origin, they are colored with blue, red, green or orange, respectively. The alignments and phylogenetic relationships were done using the MEGA X suite

In order to eliminate pollutants from contaminated water, the identification, study, and implementation of laccase-mediated processes form an intensive research area aimed at generating ecofriendly and effective tools for treating and improving water quality (Fig. 1). Laccases, which belong to the enzyme family of multi-copper oxidases (MCOs), are classified as benzenediol oxygen reductases (EC 1.10.3.2) and are also known as urushiol oxidases and *p*-diphenol oxidases [15, 16]. They are considered versatile enzymes capable of oxidizing a large number of phenolic and non-phenolic molecules due to their low substrate specificity, using oxygen as electron acceptor and generating water as a by-product [17–19]. Laccases are widely expressed in nature; they can be obtained from various fungi, plants, bacteria, lichen, and insects (Fig. 2), with laccases from each species exhibiting particular catalytic characteristics and sequences [20–22]. UniProtKB search results for “laccase” with sequence sizes between 220 and 800 amino acids, revealed approximately 7300 cellular-organism sources, with 1026 bacteria, 6258 eukaryotes, and 16 halobacteria (archaea). Hence, it can be predicted that this large number of enzymes produced by different organisms could have a wide range of applications in water bioremediation (Fig. 2). To date, many of these enzymes have been applied in processes like electrocatalysis, delignification, and ethanol production [23]. In this review, we aim to describe laccases from different organisms, used in water bioremediation, their varying properties based on their origin, their biotechnological prospectives for pollutant degradation (fabric discoloration, herbicide degradation, and emerging pollutants transformation), and the different strategies that have been explored to increase their activity and application.

## Sources of laccases that are useful in water bioremediation

### Fungal laccases

The first fungal laccase was reported by Bertrand [24], who observed that this enzyme was responsible for the color change in mushrooms of the *Boletus* genus when in contact with air. A large number of fungi have been confirmed as laccase producers, with white rot fungi being the most recognized. Among fungal species, the basidiomycetes, specifically *Agaricus bisporus*, *Pleurotus ostreatus*, *Trametes versicolor*, *Phanerochaete chrysosporium*, and *Coprinus cinereus*, produce various laccase isoforms (Table 1) [23, 25, 26].

**Table 1:** Application of some interesting fungal laccases that degrade different compounds and may be useful in water treatement

Laccase source	Applied enzyme form	Type of culture, ingredients and enzyme form	Application	Reaction parameters	Results obtained	Main putative mechanisms involved	References
Pharmaceutical compounds							
*Pycnoporus sanguineus* CCT-4518	C CI	The fungus was grown in PDA solid medium for 7 days at 28 °C. Laccase extract was produced in 50 mL of liquid media, at 28 °C for 72 h and supported	Laccase removal of 17-alpha-ethynilestradiol (EE2)	Free and immobilized laccase extract (100 U/L) were mixed with 10 mL of EE2 at 10 mg/L, 10 mL of acetate buffer, pH 4 or 5 or 10 mL of distilled water, all of this at 28 °C	80% of removal of EE2 after 24 h by the free and immobilized laccase extract at pH 4 and 5. The immobilized form had three cycles of reusability with high transformations	The laccase is able form dimers of the EE2 by polymerization of it	[49]
*Pycnoporus sanguineus*	C	The *Theobroma grandiflorum* AW was used as *Pycnoporus sanguineus* laccase (Lac) inducer, cultivated for 7 days at 28 ± 2 °C and 150 rpm	Degradation of estrogens tested	100 U/L of laccases, with 17-α-ethinylestradiol at 10 μg/mL, and 1% of inducer by 24 h	Removal 96% of estrogens after 8 h of reaction	They suggest the degradation product, with hydroxylation of estrogens	[56]
*Trametes versicolor*	F	Commercial laccase powder from *T. versicolor* (activity ≥ 0.5 U/mg) from Sigma-Aldrich	Degradation of PhAC: diclofenac, trimethoprim, carbamazepine, and sulfamethoxazole	Selected PhAC concentrations were added to the enzyme solution in individual beakers. The beakers were incubated on a rotary shaker for 48 h at 80 rpm and 25 °C	The results of this study revealed that laccase can effectively degrade diclofenac (100%), trimethoprim (95%), carbamazepine (85%), and sulfamethoxazole (56%)	Not reported	[50]
*Trametes hirsuta*	C	It was grown on PDA medium for 5 days at 28 °C and then on petri plates, pH 5 in static condition for 10 days, on Kirk’s medium. The supernatant was used	Degradation of chloramphenicol (CAP)	Different mediators like syringaldehyde, naphthol, vanillin and ABTS were added at 0.25, 0.50, 1, 3, 5 and 10 mM, to the reaction with 100 U of laccase enzyme and 10 mg/L of CAP in 0.1 M acetate buffer pH 5, by 48 h	The laccase enzyme degraded 0.5 mg/L CAP within 7 days without mediators and was efficiently degraded in the presence of laccase mediator system (syringaldehyde, vanillin, ABTS and α-naphthol)	Dehalogenation and oxidation of CAP by laccase to form chloramphenicol aldehyde which was non-toxic to the microorganisms studied	[53]
*Trametes versicolor*	C	SF (500 mL) with 20 g of dried apple pomace, Tween 80 (0.1%) and moisture of 75% (w/w), inoculated with mycelia by 14 days, 30 °C with 200 mL. Enzyme from supernatant extract	Chlortetracycline (CTC) degradation	CTC at 2 mg/L, laccase dose at 0.5 IU, pH 4.5 or 6.0, and ultrasonication	60% of CTC, considered as a recalcitrant pollutant, was removed in 2 h by ultrasonication and assisted laccase at pH 6.0. While at pH 4.5, 80% of CTC was degraded, resulting non estrogenic by products	Oxidation of C–C and C–O bonds	[51]
*Pleurotus ostreatus*	FP	PDA medium at 25 °C, and added ciprofloxacin (CIP: at 100, 200, 300, 400 and 500 ppm). The enzyme was secreted	Degradation of ciprofloxacin (CIP)	Fungi growth by 14 days with 100, 200, 300, 400 and 500 ppm of CIP	Antibiotic degradation of about 68.8, 94.25 and 91.34% was estimated after 14 days of incubation at 500 ppm CIP	Not reported	[52]
*Pycnoporus sanguineus* CS43^f^	F	STR of 10 L with 36.8% tomato juice medium, by 15 days, induced with CuSO_4_ and soybean oil at 48 h. LacI and LacII were purified	Degradation of endocrine disrupting chemicals (EDCs): nonylphenol and triclosan (a biocide)	EDC at 10 ppm final concentration were prepared in pH 5 McIlvaine buffer with 100 U/L laccase. Samples were tested every 30 min for 8 h at 25 °C	More than 95% removal after 8 h of treatment with 100 U/L at pH 5	Enzyme-driven oxidation	[41]
Plastics, personal care and herbicide compounds							
*Pycnoporus sanguineus* (CS43)	CI	11-days cultures in 10-L STR in complex liquid medium at 28 °C. Crude extract enzyme immobilized	Degradation of emerging endocrine disruptor (bisphenol A)	800 μL McIlvaine buffer (pH 3), 100 µL of ABTS (5 mM, 1.0% w/v) and 100 µL of laccase extract of *P. sanguineus* (CS43)	100% degradation of bisphenol A (20 mg/L) was achieved in less than 24 h	Probably degradation ends in the formation of 4-isopropenylphenol	[42]
*Trametes versicolor* BAFC 2234	MI	7-days cultures in 30-L STR with complex liquid medium (50% tomato juice). Purified enzymes	In vitro oxidation of phenol	The reaction mixture in 1.5-mL contained dissolved phenol (0.5 mM), 50 mM sodium citrate pH 4.5 and 0.1 U/mL laccase	84% phenol removal in 4 h. Dark colored products partly precipitated were found	Oxidative coupling of phenoxy radicals as major pathway of phenol conversion	[43]
Recombinant laccase from *Trametes sanguineus* in *Trichoderma atroviride*	F	Cultures grown in 50 mL, incubated for 4 days at 28 °C/150 rpm. Purified laccase	Degradation of xenobiotic compounds (phenanthrene and benzo[α]pyrene)	Phenanthrene and benzo[α]pyrene were added into supernatants up to at 10 ppm, incubated at 28 °C and shaken at 150 rpm for 24 h	57.5 U/L of laccase in supernatant removed phenanthrene and benzo[α]pyrene (97 and 99% respectively) present in wastewater from a biofuel industry plant	Not reported	[67]
*Nicotiana tabacum* expressing a laccase from *Pleurotus ostreatus*	C	Plants were grown for 16 days in a growing chamber at 24 °C under a photoperiod of 16:8 h (light:darkness). Enzyme secreted into rhizosphere	Phytoremediation of phenol content from olive mill wastewaters	Laccase activity of transgenic root exudates was evaluated by oxidation of 2 mM ABTS at 420 nm in 0.1 M citrate buffer pH 3.0 at 25 °C	Transgenic tobacco plants cultivated in a hydroponic solution with olive mill wastewaters were able to reduce the total phenol content up to 70%	Not reported	[92]
*Anthracophyllum discolor*	MI	It was grown in Kirk liquid medium with Tween 80 or soil supplemented with Tween 80 and wheat grains. Whole cultures	Degradation of polycyclic aromatic hydrocarbons (PAH)	Cultures and 50 mg/L of PAH at 30 °C by 28 days. 10 g soil and 0.5 g wheat grains in 30 mL tubes contaminated with a 50 mg/kg of PAHs at 30 °C by 60 days	54 up to 75% removal of phenanthrene, anthracene. fluoranthene, pyrene and benzo (a)pyrene in soil with *A. discolor*	Products of degradations were anthraquinone, phthalic acid, 4-hydroxy-9-fluorenone, 9-fluorenone and 4,5-dihydropyrene	[39]
*Trametes pubescens* CBS 696.94	C	1L SF with synthetic liquid medium supplemented with dry coffee husk. 23 days static incubation at 30 °C. Crude extracts filtered	Biodegradation of a mixture of 2-chlorophenol (CP), 2,4-dichlorophenol (DCP), 2,4,6-trichlorophenol (TCP), pentachlorophenol (PCP)	Degradation of CPs during 8 h at 40 °C, 200 rpm in flasks containing 100 mL of a CP mixture, with 15 mg/L of each CP in 50 mM phosphate buffer, pH 6.0. Enzymatic extract (5 mL) and 10 U/L	Biodegradation of 100%, 99%, 82.1% and 41.1% of CP, DCP, TCP and PCP, respectively, after 4 h. The reduction in chlorophenols, allowed 90% reduction toxicity	Not reported	[44]
*Neosartorya fischeri*	C	50 mL SF with modified Czapek medium and 20 mg of asphaltenes as carbon source, at 37 °C 100 rpm, 4 weeks. Whole cultures	Metabolization and mineralization of asphaltenes (recalcitrant petroleum fraction)	Asphaltene mineralization was quantified by measuring CO_2_ production. Cell-free extracellular medium was solvent extracted and analyzed by GC–MS	After 11 weeks of growth, the fungus metabolize 15.5% of the asphaltenic carbon, including 13.2% transformed to CO_2_	Generation of oxidized metabolites such as hydroxypyrenedione and hydroxyphenylacetic acid	[40]
*Coriolopsis rigida* LPSC 232	C	15-days liquid cultures in modified Czapek Dox medium (0.5% peptone and 0.15 mM Cu^2+^)	Detoxification of water soluble fraction from ‘‘alpeorujo” (WSFA)	Reaction mixtures containing WSFA 20% (v/v) and 20 U laccase were incubated 24 h at 28 °C and 150 rpm	Reduction of free phenols from the WSFA	Oxidation of free phenols, resulting in radical formation, leading to polymerization as well as detoxification	[208]
*Trametes villosa*	C F	Extracted and purified enzyme (Novozymes)	Bisphenol A (BPA) degradation	2.2 mM BPA incubated for 1 h with 1.0 unit/mL of laccase. The reaction mixture: 0.5 mM ABTS, 0.1 M sodium acetate, pH 5.0, and an enzyme in a total volume of 1.0 ml was incubated at 37 °C	BPA was degraded by a laccase, which was extracted and purified from DeniLite, a Novozymes’ product. Transforming and important endocrine-disturbing compound	BPA was metabolized to two compounds: one with high molecular weight due to oxidative condensation, and another identified as 4-isopro-penylphenol	[54]
Dye-based pollutants							
*Pleurotus ostreatus* URM 4809	C	250-mL SF with 50 mL of effluent with 0.05 g/L Remazol Brilliant Blue R and 10^7^ spores/mL and incubated	Decolorization dyes used in the textile industry	Cell in a microbial fuel cell with continuous laccase synthesis; and 0.05 g/L of anthraquinone remazol brilliant blue R dye	Laccase promoted decolorization by 86% of the anthraquinone dye remazol brilliant blue R (used in the textile industry)	Not mentioned, but phytotoxicity results showed that the process did not generate detectable toxic products	[46]
*Ganoderma lucidum* E47 strain	C	Solid-state fermentation in MYSA medium, pH 5.5, kept in darkness for 7 days at 25 °C. Supernatants was used as enzyme preparation. 5 compounds were tested in 0.5 L minireactor simulating an effluent	Decolorizing xanthene, azo and triarylmethane dyes	0.1 mM of organic dyes: Bengal rose; blue black naphthol; congo red; methyl orange; bromocresol green; bromocresol purple; bromophenol blue; and phenol red, 550 nm; 100 mM potassium acetate buffer pH 4.8, 5% butyl acetate, 25 °C	The best activity-stability reached in pH 4.8 at 37 °C, decolorizing xanthene, azo and triarylmethane dyes, with selectivity on bromocresol green and bromocresol purple. Activity on effluent biotreatment	Not reported	[47]
*Oudemansiella canarii*	C	SF, mycelial from petri dishes were incubated without agitation under air at 28 °C and in the absence of light by 14 days. Extract was dialyzed and partially purified	Decolorization of congo red	50 mM acetate buffer (pH 5.5) in 250-mL 140 SF with 50 mL and containing 50 mg/L of Congo red and native 141 laccase (5 U). The mixtures were incubated at 30 °C in the dark in a rotary shaker at 100 rpm	5 U were able to decolorize 80% of 50 mg/L Congo red within 24 h at 30 °C and pH 5.5	Laccase acts not only on the dye chromophore group, but also that it cleaves different covalent bonds, causing an effective fragmentation of the molecule	[48]
*P. pastoris* or *A. thaliana* expressing Lcc9 from *Laccaria bicolor*	F	SF in BMGY medium at 28 °C, the cells were suspended in of BMMY. Methanol was added to 1% every 24 h	Decolorization of triphenylmethane dyes, employed in industrial dyeing processes	The reaction mixture for the decolorization assay contained 0.1 mM of crystal violet, McIlvaine buffer and 50 μL of the enzyme in a total of 200 μL. ABTS, as the mediator, was added if necessary. Incubated in dark for 24 h	In the presence of ABTS, the decolorization rates of Crystal violet by laccases in *P. pastoris* or *A. thaliana* reached 90.7% and 83.6%, respectively	Not reported	[68]
Recombinant laccase (Lcc IIIb) from *Trametes versicolor* expressed in *Yarrowia lipolytica*	C	Cultures grown in optimized PPB medium pH 7.0 at 2 L STR	Decolorization of pollutant dyes: bromocresol purple, safranin, malachite green, kristal violet, bromothymol blue, nigrosine and phenol red	Reaction mixture was composed of 10 µL of supernatant and 90 µL of a buffer prepared by dissolving 0.1 mg of each dye in 1 mL of citrate buffer at pH 3. Dye decolorization was followed spectrophotometrically	The dye decolorization rates after the first hour were 43%, 54%, 55%, 49%, 56%, 53% and 37% for bromocresol purple, safranin, malachite green, kristal violet, Bromothymol blue, nigrosine and phenol red, respectively	Not reported	[71]
Recombinant LCC3 from *Trametes trogii* BAFC 463 in *Pichia pastoris*	C	4-days liquid cultures induced with methanol	Synthetic dye decolorization	50 µM of dye, citrate–phosphate buffer pH 4.5 at 30 °C, 1–10 U/mL laccase. Mediators used ρ-coumaric acid, HBT, violuric acid (200 µM) acetosyringone (10–200 µM)	50–100% decolorizing ability of azoic, indigoid, triarylmethane, and anthraquinonic with acetosyringone within 2 h incubation at pH 6, 70 °C	Decolorization effectiveness depended on the chemical characteristics of redox mediators and dyes, and the ratio	[70]
*Trametes trogii* BAFC 463	C F	22-days static liquid cultures in glucose (20 g/L), asparagine (3 g/L) medium with 1 mM Cu2+	Decolorization of synthetic dyes	19.5 U laccase per reaction, in test tubes at 30 °C with sodium acetate buffer (10 mM, pH 4.5) in a total volume of 3 mL. The effect of different salts, heavy metals, reaction temperature, pH and redox was analyzed	Laccase decolorized 85% of indigo carmine, xylidine, malachite green, gentian violet, bromophenol blue, 65% of fast blue RR and 30% of Azure B and Methylene Blue in 24 h	Direct oxidation of certain dyes and/or by the LMS	[58]
*Trametes versicolor*	FP F	The fungus was maintained on 2% malt agar slants at 25 ^°^C Commercial purified enzyme (Fluka)	Biodegradation of triphenylmethane dyes	Reactions in SF with 100 mL dye solution (150 mg/L) buffered with 1.6 mM 2,2-dimethyl succinate, pH 4.5, at 25 °C (laccase 1225 U/L), HBT (10^−3^ M)	Degradation dye brilliant green1 and acid green 16. resulting benzoic acid and diethylamine and 5,7-disulfo-2-naphtoic acid respectively	Oxidation of the methyl carbon of dye structure, giving stable products	[57]
*Aspergilus* expressing a laccase from *Myceliophthora thermophila*	I	Submerged fermentation of a recombinant *Aspergillus* sp. A commercial formulation, DeniLite II S, from novozymes A/S covalently immobilized	Decolorization of synthetic dyes	20 U/mL of immobilized laccase or 0.5 g in a FBR. Several dyes at 0.02% (w/v) 30 °C 0.1 M sodium acetate buffer (pH 4.5) 90 rpm	The anthraquinonic dyes acid blue 25 and acid green 27 were decolorized. The RBBR and the diazo RB-5 were only decolorized with laccase/HBT, 31 and 60%, respectively, after 24 h	Direct oxidation of certain dyes and/or by the LMS	[45]
Recombinant lcc1 gene from *Trametes trogii* in *Pichia pastoris*	F	SF at 30 °C and STR 2 L at 25 °C cultures in phosphate buffered minimal methanol (BMM), supplemented with yeast extract or casaminoacids	Decolorization dyes (amaranth, carmoisine, cochineal red, sunset yellow, patented blue, blue indigo and alizarin red S	1 mL (0.05 mg/mL of dye in 0.1 M sodium phosphate buffer, pH 5.0) and 1 IU of laccase with or without 1 mM redox mediator 1-hydroxybenzotriazole or violuric acid at 25 °C, plus dyes	All the dyes were decolorized up to 60% percent after 2 h with containing 1 U of Lcc1 and the redox mediator violuric acid 1 mM	Generation of a phenoxy radical resulting in the cleavage of azo linkages with nitrogen release	[62]

*F* free purified enzyme, *I* immobilized purified enzyme, *FP* fungal pellets, *C* crude extract or culture supernatant, *CI* crude extract immobilized, *MI* mycelium immobilized, *STR* stirred-tank reactor, *FBR* fixed-bed bioreactor, *PhAC* pharmaceutically active compound, *SF* shake flask, *PDA* potato dextrose agar, *LMS* laccase-mediator system, *HBT* hydroxybenzotriazole, *EE2* 17-alpha-ethynilestradiol, *CAP* chloramphenicol, *CTC* chlortetracycline, *CIP* ciprofloxacin, *SFMZ* sulfamethoxazole, *EDCs* endocrine disrupting chemicals, *AzBTS-(NH4)2* 2,2′-azino-bis(3-ethylbenzothiazoline-6-sulfonic acid) diammonium salt, *ABTS* diammonium 2,2′-azino-bis(3-ethylbenzothiazoline-6-sulfonate), *PAHs* polycyclic aromatic hydrocarbons, *CP* 2-chlorophenol, *DCP* 2,4-dichlorophenol, *TCP* 2,4,6-trichlorophenol, *PCP* pentachlorophenol, *BPA* bisphenol A, *WSFA* water soluble fraction from ‘‘alpeorujo”, *BMGY* buffered glycerol-complex medium

Fungal laccases are involved in sporulation, pigment production, fruiting body formation, stress defense, plant pathogenesis, and lignin degradation [27, 28]. Although most purified laccases are extracellular enzymes, wood-rotting fungi also contain intracellular laccases. It has been suggested that the localization of laccase is probably connected with its physiological function and determines the range of available substrates [29]. Laccases exist in a variety of structures; most of them are monomeric, but some are also present in homodimeric, heterodimeric, and multimeric forms. Their molecular mass ranges from 50 to 140 kDa, depending on the organism, although a typical fungal laccase will range from 60 to 70 kDa with an isoelectric point around pH 4.0 [29, 30]. Fungal laccases are usually glycosylated, with a 10–25% increase in mass, although some laccases present with a > 30% increase. The carbohydrate portion of laccases has been demonstrated to ensure their conformational stability and to protect the enzyme from proteolysis and inactivation by radicals [31, 32].

The redox potential (*E*°) of laccases has a direct relationship with the energy required to remove an electron from the reducing substrate, constituting one of the fundamental characteristics of these enzymes [33]. Therefore, laccases with a high *E*°, like fungal laccases, are of special interest in biotechnology cause they are capable of oxidizing substrates with high *E*° (*E°* > 400 mV) [33–36]. For example, the *E*° of bisphenol A (BPA), *p*-nonylphenol and azo dye BR114 are above 600 mV [37, 38]. Fungal laccases aid bioremediation through the oxidation of polycyclic aromatic hydrocarbons (PAHs) [39, 40], plastics and phenolic compounds [41–44], dyes [44–48], and the degradation of pharmaceutically active compounds [49–52], among others (Table 1). Given that laccases from white-rot fungi have the potential for phenolic compound degradation, different studies have involved the immobilization of microorganisms, such as *T. versicolor*, into silica-alginate and loofa sponges as supports for phenol removal [43]. While crude extract from *Trametes pubescens* has been used for the degradation of chlorophenols (Table 1) [44]. Also, crude extract from the white-rot fungus *Trametes hirsuta*, proved capable of degrading chloramphenicol (one of the most persistent micro-pollutants in pharmaceutical wastes), with or without mediators (Table 1) [44, 53]. Fukuda et al. [54] used a free purified laccase from *Trametes villosa* to degrade BPA, another hazardous pollutant discharged into rivers and seas, without the requirement of mediators. Meanwhile, Barrios-Estrada et al. [42] reported that the degradation of BPA (20 mg/L) occurred within the first 24 h when using *Pycnoporus sanguineus* (CS43) and *T. versicolor* laccases immobilized onto ceramic membranes (Table 1). Different steroidal estrogens can be removed or degraded from aqueous systems by the free laccases from *P. sanguineus* or laccases from *T. versicolor* or *Myceliophthora thermophila* that have been immobilized onto ceramic membranes (Table 1) [55, 56]. Other problematic compounds in effluents of textile and paper industries include synthetic dyes, of which, many are toxic for mammals. Therefore, efforts have been made towards their elimination from industrial wastewaters using laccases from *T. versicolor* and *Trametes trogii* (Table 1) [49, 58].

The laccase yield from native fungal sources fails to meet the industrial need, as natural hosts often produce several laccase isozymes making it challenging to isolate the laccase of interest, more so when the enzyme is silent or not abundantly expressed. Therefore, heterologous laccase expression has become a promising alternative [23, 59–71]. Heterologous expression of many fungal laccases has been reported in bacteria such as *E. coli* [60], yeasts like *Pichia pastoris* and *Yarrowia lipolytica* [43, 61–64], filamentous fungi such as *Aspergillus oryzae*, *A. niger*, and *Trichoderma atroviride* [65–67], and plants like *Arabidopsis thaliana* and *Zea mays* (Table 1) [68, 69]. Yeasts and filamentous fungi are usually more attractive hosts for heterologous protein production owing to their faster microbial growth, ease of gene manipulation, their ability to secrete large amounts of proteins into the growth medium, as well as the ability to perform post-translational modifications [30, 49].

Recombinant fungal laccases have also been widely applied for bioremediation purposes. For instance, the recombinant proteins Lcc1 and Lcc3 from *T. trogii*, produced in *P. pastoris* proved to be a useful biocatalyst for the oxidative degradation of several polluting dyes, such as indigo carmine, the most important dye used for manufacturing blue jeans [62, 70]. Moreover, Darvishi et al. [71] expressed and produced a recombinant laccase (Lcc IIIb) from *T. versicolor* in *Y. lipolytica,* proving its capability of decolorizing five phenolic azo dyes with > 40% efficiency after 4 h (Table 1). Similarly, Wang et al. [68] expressed a laccase from the ectomycorrhizal fungus *Laccaria bicolor* in *P. pastoris* and *A. thaliana*, which proved capable of decolorizing > 80% of the crystal violet dye, tested using laboratory-scale studies, providing an alternative to the decolorization of industrial wastes. In another study, Balcázar-López et al. [67] expressed a laccase from *P. sanguineus* in the filamentous fungus *T. atroviride*; the heterologously expressed laccase maintained similar properties to those of the native enzyme, although the recombinant showed the potential to remove > 90% of the phenanthrene and benzo[α]pyrene present in wastewater from a biofuel industry plant using laboratory-scale studies [67].

### Plant and insect laccases

The first identified and reported laccase from plants was from the Japanese lacquer tree *Toxicodendron vernicifluum* (*Rhus vernicifera*) [72]. However, studies on plant laccases are rare. Plant laccases share their molecular architecture and reaction mechanisms with fungal laccases. In general, they have a lower *E*° like bacterial laccases (0.41 V for *R. vernicifera* and a pI between 7.0 and 9.6) [22, 31, 73]. These proteins show a higher glycosylation pattern (22–45%) [74, 75], consist of 500–600 amino acids, and weigh approximately 60–130 kDa [31]. Plant laccases have been described and associated with biosynthesis and polymerization of lignin [76, 77], elongation [78–80], and the stress response [81–83].

Although plant laccases have not been largely involved in bioremediation, some applied cases have been reported. Wang et al. [84] presented a system of phytoremediation *ex planta* based on the overproduction in *A. thaliana* of a secretory laccase (LAC1), which was natively expressed in the roots of *Gossypium arboreum*. LAC1 expression in *A. thaliana* conferred resistance to several toxic phenolic compounds, probably attributable to LAC1-induced transformation. Recombinant LAC1 plants were resistance to phenolic compounds under greenhouse conditions, helping to detoxify their growth environment [84]. Watharkar et al. [85] showed that laccases and other enzymes from *Asparagus densiflorus* could be applied in the treatment of industrial textile effluents. For lab scale studies, they used a vertical subsurface flow phytoreactor based on vertical percolation of wastewater through layers of soil, root zone and a netted bottom. For large scale studies, they planted beds of *A. densiflorus* on a high rate transpiration system (HRTS), which has been used successfully for some industries. In both cases, *A. densiflorus* showed the ability to degrade dyes and reduced levels of toxic heavy metals.

Laccases from other plants have been proposed and successfully tested for dye degradation using suspension cells and purified laccases [86–90]. Huang et al. [91] identified laccases in rice (*Oryza sativa*), possibly involved in atrazine and isoproturon (herbicides) catabolism or detoxification. The two *Oryza sativa* laccases expressed heterologously in *P. pastoris*, led to the increased resistance of cells to atrazine and isoproturon, suggesting that some of the laccases could be involved in detoxification or degradation of these herbicides [91].

Plants have been successfully used as recombinant expression systems of fungal and plants laccases. Chiaiese et al. [92] expressed a laccase from *P. ostreatus* in *Nicotiana tabacum,* capable of reducing 70% of the total phenol content from olive mill wastewaters (Table 1). Other authors have expressed fungal laccases with industrial applications in rice-based [93] and tobacco plants [94, 95], as well as maize seeds [69]. Conversely, insect laccases have been reported to play an important role in cuticle sclerotization and pigmentation, as well as other processes such as wound healing and immune system development and maintenance [96, 97]. To the best of our knowledge, no insect laccase has been reported for bioremediation processes.

### Bacterial laccases

Laccase activity in bacteria was detected for the first time in *Azospirillum lipoferum*, isolated from a rice rhizosphere in 1993 [98]. Several laccases were then gradually discovered in bacteria from different genera, such as *Bacillus*, *Streptomyces*, *Klebsiella*, *Pseudomonas*, *Yersinia*, *Proteobacterium*, and *Marinomonas*, among others (Table 3) [99, 100]. Moreover, these enzymes have also been found in microorganisms of the Archaea domain such as *Haloferax volcanii* [101].

Under native conditions, bacterial laccases are involved in pigmentation processes, morphogenesis, toxin oxidation, and protection against oxidizing agents and UV light [100, 102]. The molecular weight of these enzymes is in the range of 50–70 kDa, with a majority being monomeric intracellular proteins, except those from bacteria in the *Streptomyces* genera and some other examples [103–106], such as the laccase produced by *Bacillus tequilensis* SN4, an extracellular enzyme [104].

**Fig. 3:**
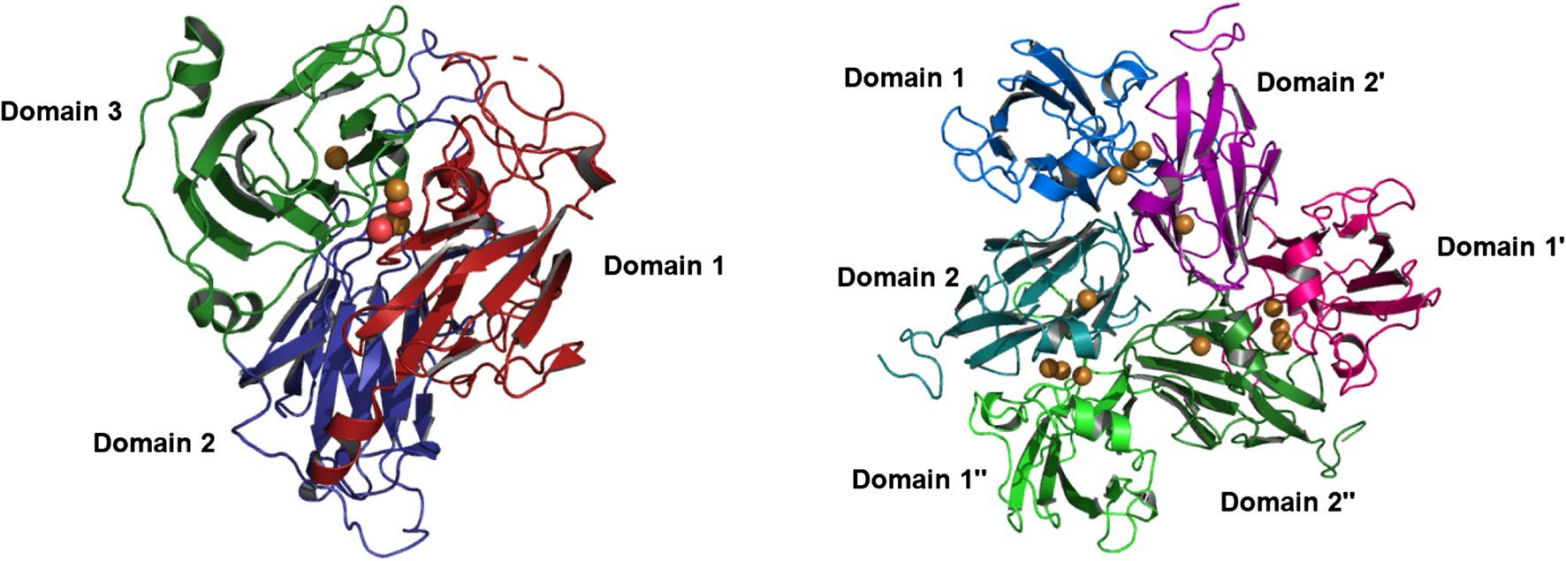
Cartoon structures of the three-domain laccase from *Bacillus subtilis* (PDB 1GSK) and the homotrimeric two-domain laccase from *Streptomyces coelicolor* (PDB 3CG8). The domain assignations were made using the SWORD partition algorithm

One of the most well-known bacterial laccases is the outer endospore coat protein CotA from *Bacillus subtilis,* which has three cupredoxin domains (Fig. 3) [107]. Other similar bacterial MCOs include the copper homeostasis protein CueO from *E. coli* [108]. Bacterial laccases with three-dimensional structures of two-domain laccases have been found in *Streptomyces, Amycolatopsis,* and *Nitrosomonas*, belonging to the group denoted as SLACs (small laccases). The implication of the absence of this domain is the need to form a homotrimer to be catalytically active (Fig. 3) [109–111].

The most significant biochemical properties of bacterial laccases are their stability under various conditions of pH, temperature, organic solvents, and salt concentrations [105]. Usually, bacterial laccases are highly stable at elevated temperatures, as seen in the *B. subtilis* laccase at 70 °C, with a thermal half-life (t_1/2_) of 250 min, or the t_1/2_ of 30 min at 80 °C of the *Streptomyces viridochromeogenes* laccase, compared with the 10 min t_1/2_ of *Cerrena unicolor* fungal laccase at the same temperature [112–115]. With respect to media pH, bacterial laccases usually work better in neutral to alkaline pH, similar to plant laccases, but unlike fungal laccases, which have optimum activities in acidic pH. Nevertheless, its optimal pH is dependent on the substrate. For instance, for phenols, such as 2,3-dimethoxyphenol, the optimal pH for *B. subtilis*, *B. clausii*, and *Streptomyces coelicolor* are pH 7, 8 and 9, respectively, while for ABTS, all three enzymes require a pH of 4 [112, 116]. However, bacterial laccases have shown greater tolerability to high concentrations of sodium chloride, being active in 1 M or higher concentrations, as seen with the laccases of *Marinomonas mediterranea* and *Bacillus halodurans*, among others [115, 117]. Some bacterial laccases have exhibited high tolerance to different solvents, including ethanol, methanol, dimethylformamide, acetonitrile, acetone, and dimethylsulfoxide, as observed in the *Bacillus pumilus* W3 laccase, which generally retains > 50% of its activity in solvent–water mixtures [118].

Although bacterial laccases are generally more robust and stable enzymes in comparison to fungal laccases, their application has been restricted by their low *E*° (*E°* T1 < +460 mV) [22, 112]. Nevertheless, bacterial laccases represent a good option for the treatment of contaminated wastes such as textile effluents, which usually have high salt concentrations (40–100 g/L) and alkaline pH [119].

Heterologous overexpression of bacterial laccases has been reported in *E. coli* [99, 112, 115, 118, 120–125], *P. pastoris* [126–128], and *Streptomyces coelicolor* [116]. Although *E. coli* is the most used expression system for bacterial laccases, the production of MCOs in its cytoplasm has a major drawback as its copper homeostasis systems maintain a cellular copper concentration around 10 µM under aerobic conditions [129–131], which is insufficient to achieve fully loaded copper laccases [132]. Copper-depleted laccases are incapable of reaching their maximum catalytic activity [122, 132]. This limitation can be overcome by changing the oxygen concentration when cultivating recombinant *E. coli* expressing laccases, because under anaerobic (or microaerobic) conditions, the intracellular copper accumulation is 80-fold higher, compared with that attained under aerobic conditions [103, 122, 132, 133].

Bacterial laccases have been used in bioremediation, mainly for the degradation of synthetic dyes. Liu et al. [99] reported a thermostable and pH-stable *Klebsiella pneumoniae* laccase which degrades diverse dyes used in industrial processes (such as reactive brilliant blue X-BR, reactive dark blue M-2GE, congo red, bromophenol blue, and malachite green, among others) in short reaction times (90 min) under diverse pH values at 70 °C (Table 3). Another case is the *B. pumilus* CotA-laccase mutant WLF, obtained by Luo et al. [125], which has an improved expression in *E. coli* and has been tested for the degradation of diverse dyes, obtaining higher decoloration yields with anthraquinonic and triphenylmethane dyes, compared with aromatic heterocyclic dyes (Table 3). Meanwhile, high decolorization of toluidine, malachite green, and reactive black 5 by the azide-resistant spore laccase from halotolerant *Bacillus safensis*, has also been reported [134]. Recombinant laccases from *E. coli* [116] or *Thermus thermophiles* [126] expressed in *P. pastoris*, efficiently decolorized congo red and remazol brilliant blue R (Table 3). Recently, the recombinant *Streptomyces ipomoea* SilA laccase expressed in *E. coli*, in the presence of mediators such as acetosyringone and methyl syringate, enhanced the decolorization and detoxification of a variety of textile dyes, like reactive black 5, orange II, and indigo carmine, also diminishing the toxicity of acid orange 63, tartrazine and its products [135].

Outside these pollutants, other contaminant compounds have been degraded with laccases. Singh et al. [136] used recombinant *Yersinia enterocolitica* laccase to biotransform two nonsteroidal anti-inflammatory drugs (diclofenac (DF) and aspirin), obtaining complete transformation of these molecules in 24 h (Table 3). Furthermore, DF and mefenamic acid were also transformed by laccases produced by *Streptomyces cyaneus* [137], and *Streptomyces mutabilis* laccases transformed antibiotics like sulfadiazine and sulfathiazole [138]. Similarly, the recombinant *S. ipomoea* SilA laccase expressed in *E. coli,* has shown a high percent conversion of ciprofloxacin and norfloxacin [139]. Interestingly, PAHs such as anthracene, pyrene benzo[α]pyrene, phenanthrene, and fluoranthene, have been oxidized by the recombinant laccase CotA from *B. subtilis* produced in *E. coli* [123]. Moreover, laccases from *S. cyaneus* have demonstrated full BPA degradation after 2 days [137].

## Structure of laccases and comparative structure analyses

**Fig. 4:**
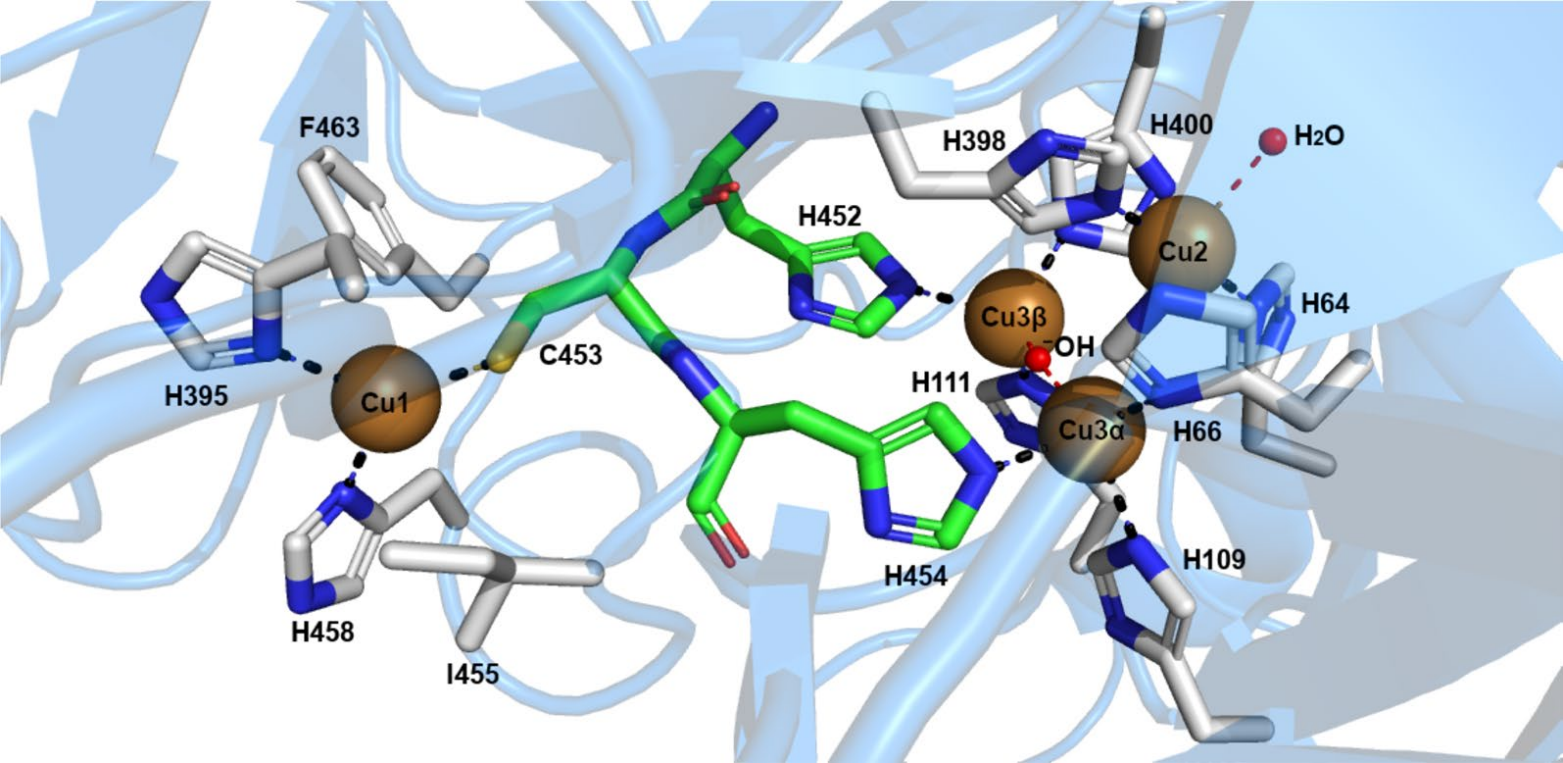
Representation of the different amino acids of the catalytic site that coordinates the catalytic coppers in *Trametes versicolor* laccase (PDB 1KYA). The amino acids of the histidine-cysteine pathway are in green

Laccases as MCOs, have four copper atoms in remarkably special oxidation states: one type-1, one type-2, and two type-3s, all forming their catalytic site (Fig. 4). Laccases are members of the cupredoxin superfamily, particularly the family of multi-domain cupredoxins. This family is characterized by the cupredoxin fold, which consists of two β-sheets arranged into a Greek-key barrel. The Greek-key motif has at least seven antiparallel β-strands twisted to form a closed barrel structure, in which some β-strands are adjacent in space but not in sequence [109].

The classification of the copper atoms is based on the environment of the metal ion and its spectroscopic characteristics; T1: paramagnetic ‘blue’ copper, with an absorbance at 610 nm, T2: paramagnetic ‘non-blue’ copper, and T3: a diamagnetic spin-coupled copper–copper pair, with an absorbance at 330 nm [140]. T1 copper has the highest *E*° and is the substrate oxidation site. This ion has a trigonal orientation, with two conserved histidines and one cysteine as equatorial ligands, and an axial ligand of variable nature, usually methionine in bacteria and leucine or phenylalanine in fungal laccases. Type-2 and the two type-3 coppers form a cluster, where molecular oxygen is reduced, and water is released. Types-2 and 3 copper atoms are coordinated by histidine side chains (T2 by two of them and T3 by six) (Fig. 4). A hydroxyl bridge maintains the antiferromagnetic coupling between T3 copper atoms [141].

Common laccases contain three homologous cupredoxin domains. Their mononuclear copper site exists in domain 3 and their trinuclear cluster is formed at the interface between domain 1 and 3 [109]. In laccases with this topology, the function of domain 2 is to join and position domains 1 and 3, enabling the formation of the trinuclear cluster [106]. In contrast, in two-domain laccases, which are from bacteria and are so called small laccases, their mononuclear copper site exists in domain 1 or 2, but for the formation of their trinuclear cluster they need to oligomerize as homotrimers, generating this catalytic site at the interface between the domain 1 of one monomer and the domain 2 of the other monomer [106, 142].

In both cases, the distance and relative position between the copper sites are conserved (the distance between T1 copper and the cluster); about 12 Å in all laccases [109].

There are several hypotheses on the evolution of laccases; all of them consider that the cupredoxin domain, with one copper atom in its structure, developed in different forms of MCOs, including dicyanin, ascorbate oxidase, nitrite reductase, ceruloplasmin, SLACs, and three-domain laccases [140, 143]. These hypotheses postulate different pathways and intermediate species that led to the development of the trinuclear cluster and the origin of the different MCOs. These structures maintain the original cupredoxin domain but are associated in dimers of independent chains or form longer chains by gene fusion [111]. Some of these domains maintain the copper-binding site, and different forms of interdomain association were evolutionarily explored by independent divergence to develop at least two cluster types of three copper atoms [142]. Other interesting schemes of this hypothesis have also been reported [144, 145].

Three-domain laccases are mainly studied in fungi, but have also been observed in some bacteria, archaea, plants, and insects [22, 146]. The database containing information on different laccases and MCOs is BioCatNet [147]. Laccases are considered “moonlighting” proteins, owing to their multiple biological activities [108]. PDB structures of > 70 fungal and a few bacterial laccases have been reported, crystallized in their wild-type, mutant, and derivative forms, as well as complexed to a variety of substrate-like ligands and oxygen reactive species [109, 148]. This set of structures has shed light on their stabilities and functional characteristics, as described above.

**Fig. 5:**
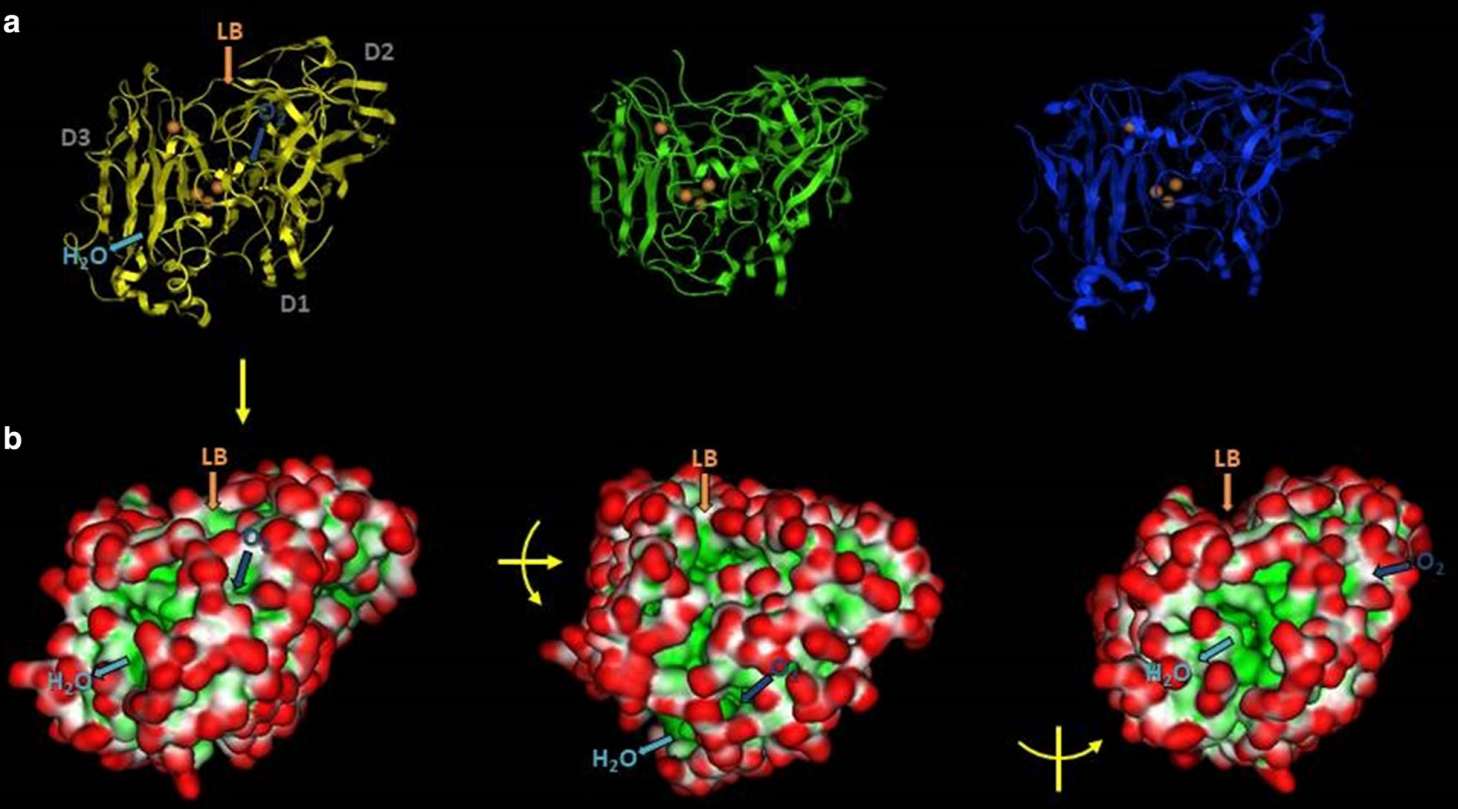
Laccase structure conservation and function. **a** Structure of *Trametes versicolor* (PDB ID 1GYC), and *Bacillus subtilis* (PDB ID 1GSK) laccases compared to *Cucurbita pepo* (zucchini) ascorbate oxidase (PDB ID 1AOZ) from left to right. Domain 1 (D1) is at the front and right of the structure, domain 2 (D2) is behind and in the upper portion, domain 3 (D3) is at the left. Brown spheres symbolize the position of copper atoms, T1 above the trinuclear cluster. **b** The molecular surface shows protruding chemical groups, in red, and concave or cavity regions, in green. Some of these latter regions correspond to the ligand-binding site (LB) along with the dioxygen molecule entrance (O_2_) and the water exit (H_2_O) channels. Central and right images were created from that on the left by rotating it 30° over the horizontal axis, or 30° over the vertical axis, respectively

Nevertheless, no structures from other species have been reported, except a plant (zucchini) ascorbate-oxidase closely homologous to laccases [149, 150]. The general three-domain structure of laccases is maintained in different species, with the loops protruding the cupredoxin domains being the most conspicuous difference [111, 151], as well as the form and by consequence, the selectivity at the substrate binding site [152]. More subtle differences are situated in the axial position of the T1 copper atom, causing the span of *E*°s from 400 mV in plant and bacterial laccases to approximately 800 mV in the majority of fungal types [22, 139, 153]. In Fig. 5, we compare the structures of representative fungal, bacterial, plant, and insect laccases. This latter structure was homology modeled from its amino acid sequence. The conserved orientation of the coppers and the cavities for substrates and products are also presented.

Several review papers have been published discussing the structure of laccase and its implications on function. For instance, the description of the molecular mechanism of substrate oxidation in the T1 site, the intramolecular electron transfer to the trinuclear cluster located about 12 Å away, and the oxygen reduction to water, can be understood in the scheme of Hakulinen and Rouvinen [109], and the detailed descriptions of Mot and Silaghi-Dumitrescu [140], Pardo and Camareno [154], and Sitarz et al. [155]. These references show the complexity and subtlety of the reaction pathway through enzyme structure. This mechanism involves a substrate binding pocket [154, 156], which confers selectivity by proper docking, and also affects the *E*° by induced fitting to the active site. There, the T1 copper atom extracts an electron from the substrate, followed by a relay of protein functional groups, namely thiol, carbonyl, and imidazole groups, which transfer that electron through the trinuclear cluster, where they are gathered until four electrons are collected. T3 copper atoms transfer such electrons to the T2 copper, and an oxygen channel allows an oxygen molecule to reach this buried metal ion and be reduced [140].

The reduced oxygen atoms are then converted to water following assistance from two carboxylate groups from aspartic and glutamic acids, which transfer the required hydrogen atoms. At least two structural water molecules also contribute to the electron transfer process. The generated water molecules finally go out a second channel formed by polar residues of the protein. Copper atoms undergo a series of at least five stages during this process [109, 154, 157]. This depiction of the process exposes the number of chemical species involved during oxidative catalysis, and the essential participation of the molecular structure [156].

Characteristics of the biological activity of laccases enable the name “green catalysts,” as they oxidize different substrates, only require oxygen molecules as reactants, and only produce water molecules as byproducts [22]. The structure of the molecular system is complex, involving its protein structure as well as its carbohydrate moiety as a stabilizing fastener [140, 156] and functional coadjuvant, along with structural water molecules, a C-terminus rearrangement, the coordination state of copper atoms, electron transfer through main and side-chains, and mediators [158]. Moreover, solvent composition is also a determinant in laccase stability, for example the presence of polyhydroxyl compounds [159].

Different approaches have been employed to handle such complexity for the development of laccases tailored to specific industrial and bioremediation processes [22, 152, 156]. These approaches can be classified as rational (computer prediction based on molecular modeling, quantum mechanics, and molecular dynamics simulations) [152, 160], semi-rational (experimental assays of trial and error mutants on a structural position identified by knowledge-based analyses or calculation), directed evolution screenings, assays of chimeric structures and laccase immobilization [152, 154, 161], and recent synthetic biology schemes [162]. These approaches have successfully produced laccase mutants or derivatives with enhanced temperature or organic solvent stability; activities tailored to develop specificity to certain substrates; higher *E*° in the T1 site, enhanced heterologous expression, the shift of pH-activity profiles, and tolerance to chemical inhibitors. In structural terms, these improvements were achieved by modifying the functional groups in the substrate binding site and T1 copper coordination [22], as well as introducing stabilizing mutants in the domain interface [163]. Nevertheless, the precise prediction of the effect of a specific mutation remains elusive [152, 155].

## Mechanism of action of laccases

**Fig. 6:**
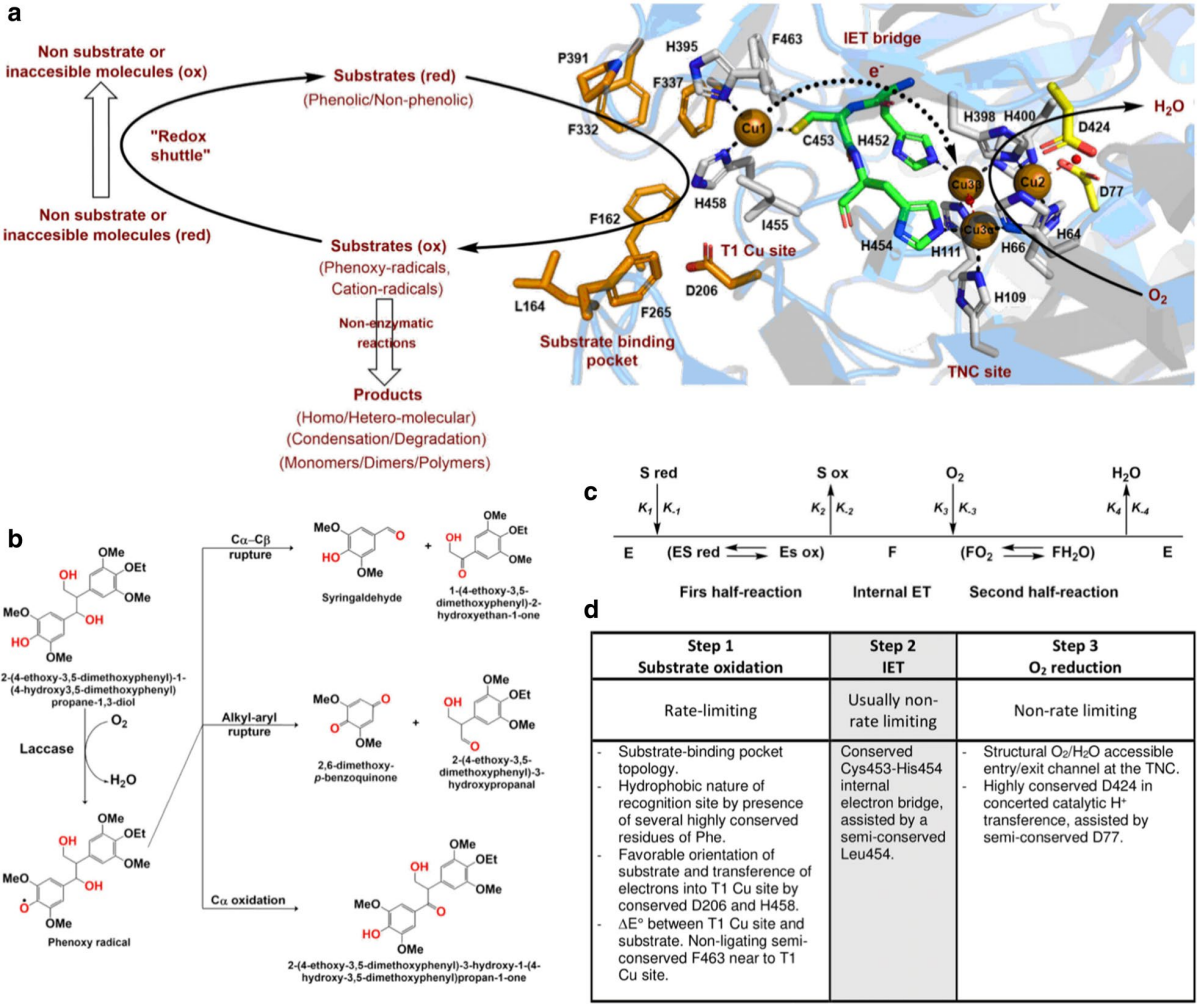
Mechanism, kinetic model and structural elements involved in laccase functional properties and reaction. **a** Representation of the laccase mechanism of action in the active site of *Trametes versicolor* laccase (PDB 1KYA). In orange are represented those aminoacids involved in the binding, stabilization and orientation of the substrate, in grey and green those that are involved in the coordination of catalytic coppers and the electron transfer and in yellow those that transfer protons for the oxygen assisted reduction. **b** Laccase action on a lignin-model illustrating the domino effect [198]. **c** Complex two-site ping-pong *bi*-*bi* kinetic model proposed for the laccase reaction [184, 185]. **d** Structural and functional elements involved in different steps of the laccase reaction

The potential application of laccases in numerous and different biocatalytic processes for industry and environmental solutions has increased the interest in understanding their mechanism of action. In general, laccases oxidize a wide range of substrates; typically substituted phenols and aromatic amines, which are transformed into free radicals (Fig. 6a) [164, 165]. Unstable chemical products and primarily generated free radicals commonly start domino reactions (Fig. 6b), leading to complex chemical transformations of biological relevance such as lignin synthesis and degradation [166].

The overall laccase reaction involves one electron (1e^−^), sequential oxidations of four molecules of reducing substrates, concurrently with two double electron (2 × 2e^−^) reductions of oxygen atoms into their respective H_2_O molecules. This process is accompanied by a catalytic exchange of 4 H^+^ equivalents [167]. From the structural, mechanistic, and kinetical points of view, a laccase reaction is approached as two half-reactions connected by an internal electron transfer (IET) step, assisted by the catalytic copper ions located at the T1 Cu and T2 Cu/T3 Cuα/T3 Cuβ trinuclear cluster (TNC) sites [157, 167, 168].

The fully conserved nature of the eleven (one Cys and ten His) residues forming the T1 copper and TNC laccase sites, and in general all MCOs, explain their essential role in the catalytic action. This relationship has been experimentally demonstrated by the comparison of sequences and mutagenic approaches in many studies [168–170]. Similarly, other fully or highly conserved residues achieving important roles in different catalytic steps involved in laccase action have been identified and include the recognition and docking of reducing substrates, IET from the T1 copper ion into the TNC site, and reduction of oxygen atoms at the TNC site. As a rule, these residues are located in the vicinity of their respective sites of action, where they appear as second sphere residues [171].

Despite these advances in the understanding of the action of laccases in terms of structure–function, a complete picture relating their molecular properties and mechanisms with their kinetic performance remains unclear. This condition could be understood based on the evolutionarily adjusted broad range of organic molecules capable of being oxidized by a laccase, and the relative ability of diverse laccases to drag substrates into recognition sites and favorably orientate them to be oxidized, limiting an integrated scheme [172–175]. A brief review of how these structural elements are mechanistically linked to the function of the catalytic copper center, and the manner in which their kinetic performance is influenced, is outlined below.

In the first half semi-reaction, 1e^−^ substrate oxidation takes place at the T1 copper site located at the bottom of the substrate binding pocket. In *T. versicolor* laccase 2 (TvL), this substrate interaction region is delimited by several highly conserved hydrophobic residues; Phe 162, Leu 164, Phe 265, Phe 332, and Pro 391, that form a favorable environment for the docking of hydrophobic molecules, such as the typical aromatic phenol/amine substrates to be oxidized by laccase. In addition, the fully conserved residue Asp 206 (near His 458 of the T1 copper site), located at the bottom of the substrate binding pocket, contributes to substrate stabilization and orientation (through O–H interactions) at the catalytic T1 copper site, through the participation of a fully conserved His 458 [174, 176]. This last residue is exposed to the solvent at the interface of the substrate binding cavity. In this manner, Asp 206 acts as an essential mechanistic element by promoting electron subtraction and transfer from substrate donor molecules into the T1 copper ion (Cu^2+^ → Cu^1+^) through a direct interaction with His 458, in the T1 copper site. Moreover, the high *E*°′ observed on this TvL has been directly related to the presence of the non-ligating semi-conserved hydrophobic residue Phe 463 at the axial position in this center [177]. Based on this, laccases are commonly classified into three classes: low *E*°′ laccases (< +460 mV), typically found in plants and bacteria, with the axial position at the T1 copper site occupied by a 4th ligating Met residue; medium *E*°′ laccases (from 460 to 710 mV), typically reported in ascomycetes and other lignin-degrading fungi sharing similar ecophysiological niches, with a non-ligating Leu residue at the axial position of the T1 copper; and high *E*°′ laccases (> +710 mV), typically reported in white-rot basidiomycetes, with the non-ligating Phe residue at this position [22, 153, 174, 178].

In the IET linking step, electrons from the reduced T1 copper ion are rapidly transferred into Cu3α at the TNC site. This step is a result of the interaction between conserved Cys 453 (linked to the T1 Cu^1+^ ion) and His 454 (linked to Cu3α), mediated by an N–H backbone group of the semi-conserved Leu 455. This structural redox-arrangement is known as the Cys-His electron transfer (ET) bridge. The second half-reaction of oxygen reduction takes place in two consecutive two-electron steps at the TNC in a redox reaction, which requires the synchronized action of the Cys-His IET bridge to complete one catalytic cycle. In this step, oxygen molecules diffuse into the TNC through the entry solvent accessible channel [179]. The first two electrons are donated by T3 copper ions of the fully reduced enzyme to oxygen, resulting in the formation of the laccase peroxide intermediate (PI). The second two electrons are then transferred from the T1 and T2 copper ions to reductively cleave the O–O bond. This step occurs in a concerted way with a catalytic transfer of H^+^ from the carboxylic Glu 424 residue, in an action that involves the mediation of the semi-conserved Asp 77 exposed to solvent at the interface of the TNC site. The reductive O–O cleavage results in the formation of the laccase native intermediate (NI). This mechanism of electrons/H+ transfer avoids the generation of reactive oxygen radicals at the TNC site, and the first H_2_O molecule produced, diffuses out of the TNC site through the exit solvent accessible channel [109, 179, 180].

Kinetic properties of laccases, *K*_m_ (apparent affinity), *k*_cat_ (catalytic rate constant), and *k*_cat_/*K*_m_ have been commonly determined in steady-state studies (Fig. 6c, d). A complex two-site ping-pong *bi*-*bi* mechanism has been suggested from a study with the pioneering model *Rhus vernicifera* plant laccase (oxygen reduction with different organic molecules as donor substrates). In addition, the rate-limiting step was associated with the organic substrate oxidation half-reaction (step 1), as the IET (step 2) rate constant (*k*IET) is higher, compared to the catalytic rate constant (*k*_cat_), and oxygen reduction (step 3) is controlled by diffusion [181]. Results of subsequent research on diverse laccases with several substrates are in accordance with this initial finding, attributing the rate-limiting step to the substrate oxidation reaction. A linear relationship between the laccase catalytic performance, referred to as log(*k*_cat_/*K*_m_) and the *E*° difference between the T1 copper site and substrate (*ΔE*°′) have also been derived from experimental data [182].

Given the catalytic versatility of laccase, novel technological tools have recently been introduced to the theoretical analyses of the molecular basis of laccase action. In recent work using several theoretical approaches, the importance of the fully conserved residues, Asp 206 and His 458, as well as the hydrophobic nature of the substrate recognition site, were confirmed by modelling with two fungal laccases (TvL and CuL). Likewise, the physicochemical properties that influence the *K*_m_ (the ionization potential, shape, and binding affinity of the substrate) were defined. General results highlight that this catalytic constant depends on substrate binding, as well as enzyme molecular characteristics [152].

## Engineering laccases: the quest for a better biocatalyst

As mentioned in other sections of this review, laccases have a broad spectrum of applications in different fields [102]. However, in some cases, the improvement of certain properties is desirable to achieve commercially attractive laccase-based applications or enhance catalyst performance. In this section, modern tools and strategies to achieve stability, optimum operation conditions, and inhibition by reaction medium components, are reviewed.

Laccase activity is lost after a certain number of reaction cycles. For instance, in the presence of free radicals produced from 1e- subtraction of phenols, aromatic amines, or mediators, the enzyme has been reported to eventually inactivate [183–185]. Although the mechanism of inactivation by free radicals is not well understood, it may involve oxidation of important residues near the active site that are essential for maintaining protein structure, as demonstrated for other enzymes catalyzing similar reactions [186]. Another important issue is the activity and stability in the presence of organic solvents; due to the hydrophobic nature of most substrates, the presence of co-solvents is necessary to increase substrate availability. Most enzymes are susceptible to activity loss in the presence of organic solvents, owing to denaturation and other mechanisms [187]. Laccases are not exempted, given their instability in the presence of methanol, ethanol, acetone, acetonitrile, as well as other water-soluble solvents.

Mesophilic proteins from organisms living in normal environmental conditions tend to be stable in aqueous media at 25–35 °C and neutral pH. Commercially available laccases come from such organisms, for example laccases from *T. versicolor*, *A. bisporus*, *P. ostreatus* and *Rhus vernicifera* can be purchased from international companies. A recent review of patents related to laccase applicability can be consulted in Ref. [188]. One strategy to overcome the limitations of mesophilic proteins is to search for those with desired properties, usually focusing on samples from extreme environments. For instance, a bacterial laccase (CotA) from *B. clausii,* was active in the presence of up to 1 M NaCl over a broader pH range, compared to CotA from *B. subtilis* [112]. Further, a novel laccase-like protein obtained through metagenome mining of samples from the South China Sea displayed halide- and alkali-tolerance, as well as the ability to decolorize dyes [189], which are properties of interest for industrial applications. Conversely, one may introduce or enhance the desired property by protein engineering. A powerful strategy is laboratory evolution and rational design, usually aided by computational tools [22, 176, 190, 191]. An example of laboratory evolution is the quadruple mutant L386W/G417L/G57F/K317N of the CotA-laccase from *B. pumilus* W3, where the mutations enhanced thermal stability and improved dye degradation [125].

The optimum pH for enzyme activity may be modified following these strategies. Based on biotechnological requirements, fungal laccases are the most attractive given their high *E*°. For most of these enzymes, the optimal pH is acidic (pH 3–5); however, some applications require the optimum to be closer to physiological conditions such as neutral pH or even alkaline conditions [102]. For instance, laccase-initiated precursor cross-coupling has been used to generate polymeric compounds for cotton dyeing; this application is limited by low precursor solubility at acidic pH, which requires alkaline conditions to be solubilized. Other uses of laccases that may benefit from enzyme function at neutral conditions are biofuel cells, biosensors, and medical applications, in which the operation conditions (such as sample pH) are close to neutrality. Examples of the modification of optimum pH of laccases are available in the literature. For instance, a laccase from *Pycnoporus cinnabarinus* was made to evolve during six cycles, using a multiscreening assay with three different substrates, at pH 5 [192]. The resulting variant, α*-3PO, contained five mutations in the mature protein; N208S, R280H, N331D, D341N, and P394H. Compared with the parent enzyme at pH 5, the variant showed a similar or higher catalytic efficiency (k_cat_/*K*_M_) towards model substrates: an 18-fold increase for ABTS (nonphenolic substrate), a 5.7-fold increase for sinapic acid (phenolic substrate), and a 1.6-fold increase for 2,6-dimethoxyphenol (DMP, phenolic substrate). Interestingly, the optimum pH shifted from 2 to 4.5 for ABTS, and from 3 to 4.5 for DMP. Another example is the in vitro evolution of the thermostable laccase mutant MtLT2 from the ascomycete fungus *Myceliophthora thermophila*; after five cycles, the optimum pH of the resulting variant shifted from 4 to 6.5 [193]. With respect to catalytic efficiency, the variant showed an increase of 31-fold for ABTS and a ninefold increase for DMP. This variant contained one conservative mutation (D530E) away from the active site on the protein surface that may be unrelated to the shift in optimum pH. Another mutation; N109S, occurred in the vicinity of the T2/T3 site, and according to the authors, may have remodeled the configuration of the site, thus conferring the trinuclear cluster a certain resistance to inhibition by OH^−^ [194]. Following a different approach, a shift of 1.4 in optimum pH was obtained after site-directed mutagenesis of a laccase from *T. versicolor*. The mutagenic site was decided following X-ray structure analyses of a complex between the enzyme and xylidine, a nonphenolic substrate [195]. Residue Asp 206 was observed to strongly interact with the substrate, and when replaced by Asn, the mutant showed an optimum pH of 4.8, compared to 3.4 for the parent enzyme. According to the authors, this shift may result from a lower deprotonation of the substrate at acidic pH in the absence of the carboxylate group, thus, requiring a higher pH in bulk water.

With respect to organic solvent stability, the thermostable laccase mutant MtTL2, from *M. thermophila,* was made to evolve using a screening assay in the presence of increasing concentrations of organic co-solvent; the activity and stability increased from 20 to 60% v/v [196]. After five cycles, a variant (R2) showing enhanced properties was selected for detailed characterization. Variant R2 displayed improved activity in the presence of co-solvents such as 50% v/v ethanol (19-fold) and 30% v/v acetonitrile (12.6-fold), compared with the parent enzyme, as well as improved tolerance to the presence of organic co-solvents. Impressively, it retained > 80% activity after a 24 h incubation period in the presence of as high as 50% v/v organic co-solvents of different natures, such as acetonitrile, dimethylacetamide, dimethylformamide, dimethylsulfoxide, acetone, methanol, and ethanol. Furthermore, it was more active in the presence of 30% v/v of acetonitrile or ethanol, compared to several native fungal enzymes, such as laccases from *T. versicolor*, *P. cinnabarinus*, *Coriolopsis gallica*, and *P. ostreatus*. Interestingly, the optimum pH shifted from 4 to 5, with variant R2 retaining 70% activity at pH 6. These improved properties could not be attributed to a higher intrinsic reactivity of the variant, as the *E*°s of the T1 and T2/T3 sites were similar to the parent enzyme. This variant accumulated four beneficial mutations; one was a conservative mutation of a residue buried in the protein matrix (L429V) and three mutations (E182K, S280N, and N552H) occurred on the protein surface, away from the catalytic site, probably establishing new interactions that could reduce the susceptibility of the protein to denaturation in the presence of organic co-solvents. In a completely different approach, a chimeric laccase was obtained through domain swapping of two laccase mutants from *Coriolopsis* sp. (OB1), and the above mentioned α*-3PO from *P. cinnabarinus* [63]. Domains 1 and 3 contain residues coordinating the copper atoms in laccase, while domain 2 bridges domains 1 and 3, and shapes the active site cavity of the T1 copper. The chimeric laccase contained domains 1 and 3 from OB1 and domain 2 from α*-3PO. The resulting enzyme was functional and displayed better tolerance to organic solvents such as 50% v/v ethanol or methanol, compared with the parent variants. Thermal stability was also enhanced in the chimeric laccase. The half-life at 50 °C was 13.8 h, compared with 8.1 h for OB1 and 3.6 h for α*-3PO.

Another property particularly relevant for commercial applications is total turnover (TTN). Total turnover is defined as the amount of converted substrate or generated product per amount of enzyme consumed in the reaction. As mentioned above, enzymes inactivate after a certain number of catalytic cycles or turnovers. Thus, TTN may be interpreted as yield, reflecting the activity and stability of the catalyst during operation, and it is independent of time. In the case of free-radical generating reactions such as phenol oxidation, laccases may inactivate due to oxidation of relevant residues. Using a fungal laccase from *C. gallica* as a model, quantum mechanics/molecular mechanics (QM/MM) calculations were performed to identify which residues are susceptible to oxidation within the active site cavity of the T1 copper; where free radicals are generated after substrate-electron subtraction [197]. According to theoretical predictions, phenylalanine residues, particularly those exposed to the solvent, are prone to oxidation. Upon replacement by site-directed mutagenesis with nonaromatic apolar residues, single mutants F357L and F413A showed similar kinetic constants with syringaldazine, but higher TTN (2- to 2.6-fold) during 4-methoxyphenol oxidation; a reaction that generates phenoxy free radicals.

In summary, manipulating the intrinsic multifactorial properties of enzymes in general and laccases in particular, is feasible, using modern protein engineering tools. Properties such as optimum pH, organic solvent tolerance, thermotolerance, and operational stability may be targeted to modify desired biocatalyst properties according to water treatment requirements.

## Potential application of laccases in water bioremediation

There are more than 15 laccase-based commercial products used in the textile, food and paper industry, which reflects the viability of their industrial use [199]. The most successful and versatile applications have been developed for the textile industry, particularly for the bleaching of indigo-stained denim, such as Denilitel, DeniliteITM, Zylite, and Bleach-cut 3S. Furthermore, the commercialization of the laccase Metzyme, which has high ligninolytic properties, opens the possibility of commercial use of other laccases in the near future [199]. Unfortunately, no commercial laccase products for water treatment are available yet.

Nevertheless, there are experiments that clearly demonstrate the efficacy of laccases to remove pollutants in real wastewater. A laccase from *Trametes versicolor* has proven to be effective at reducing estrogen concentrations from a municipal wastewater treatment plant in Rolla, Missouri (USA), tested at laboratory-scale. Because of cost effectiveness, laccases may present important advantages over other degrading enzymes in municipal wastewater treatment [200]. The fungal crude extracts from *T. pubescens* MUT 2400 transformed target molecules and decreased up to 70% of the initial concentration of 2-hydroxybiphenyl, Naproxen, DF, and diethyl phthalate under real municipal wastewater conditions in north-western Italy (Turin) [11]. Also, the positive effect of removing phenol from aqueous refinery samples was demonstrated using laccase SP504 from Novozymes (Franklinton, NC, USA) [201].

The efficacy of immobilized laccase from *Coriolopsis gallica* was demonstrated for the elimination of pollutants such as BPA, DF and 17-a-ethinylestradiol (EE2) in real samples from the AQUIRIS wastewater treatment plant [202]. While the laccase of the white-rot fungus *Coriolopsis polyzona* was immobilized onto spherical nanoparticles and tested in effluent water from the wastewater treatment plant Birsfelden, Switzerland, demonstrating that in real wastewater, immobilized laccase can retain its activity over 1 month [203]. In addition, laccase and tyrosinase as combined crosslinked aggregates showed high transformation of acetaminophen to its oligomers in real municipal wastewaters [204]. Even with the presence of colloids and certain ions or other molecules that induce the formation of precipitates that affect enzyme stability, the removal of BPA by laccase was demonstrated in a continuous enzymatic membrane reactor operated with real wastewater [205]. While, Jahangiri et al. [206] demonstrated that laccase from *Phoma* sp. UHH 5-1-03, cross-linked to polyvinylidene fluoride membranes, removed with high efficiency pharmaceutical compounds such as acetaminophen, bezafibrate, indometacin, ketoprofen, mefenamic acid, and naproxen under the harsh conditions of real wastewater [206]. Furthermore, laccase from *Trametes versicolor* that was encapsulated in core–shell magnetic copper alginate beads, allowing bead recycling with enhanced field feasibility, efficiently degraded triclosan and recalcitrant dyes in real wastewater from a chemical factory [207].

Along with the positive results however, there are inconveniences, which should be remedied to achieve commercial success. These include the improvement of the search for enzymes using omics tools for the application of microorganisms that present enhanced production of laccases, improvement in the processes of enzyme production taking into account the cost and the environmental impact, the development of scalable and economically viable processes that do not affect the environment, and in which the stability and reuse of enzymes is ensured. Added to this, as in the textile industry, customized formulations may be developed according to the treatment conditions.

In summary, fungal, plant and bacterial laccases are currently used in many bioremediation applications, removing pharmaceutical to industrial pollutants as summarized in Tables 1, 2 and 3. Manipulating the intrinsic multifactorial properties of enzymes in general and laccases in particular, is feasible using modern protein engineering tools. Thus, the generation and/or discovery of laccase variants able to function under a wide range of conditions (pH, temperature, suspended solids, mechanical stress, among others) will help in the development of robust enzymes for commercial products in the pressing issue of using water in a sustainable fashion.

**Table 2:** Application of some interesting plant laccases that degrade different compounds and may be useful in water treatement

Laccase source	Applied enzyme form	Type of culture, ingredients	Application	Reaction parameters	Results obtained	Main putative mechanisms involved	References
Dye-based pollutants							
*A. densiflorus*	P R	Single plant having a total biomass of 70 ± 4 g in 500 mL beaker having 200 mL of 20, 40, 60, 80 and 100 mg/L by 12 h in dye solution in distilled water Root tissue showed laccase (138%), lignin peroxidase (129%), riboflavin reductase (111%), DCIP reductase (47%), tyrosinase (26%) and azo reductase (18%) activities	Progressive dye accumulation and removal of Rubin GFL (RGFL) dye	Reactors (phytoreactor of 30 L) were watered with 500 mL tap water every day for 30 days	*A. densiflorus* decolorized Rubin GFL (RGFL) dye (40 gm L^−1^) up to 91% within 48 h. RGFL at 20, 60, 80 and 100 mg/L were decolorized by 91, 82, 69% and 61%, respectively	Proposed oxidative cleavage and deamination of the dyes. Phytotoxicity study demonstrated reduced toxicity of biotransformed RGFL	[85]
*Tagetes patula*, *Aster amellus*, *Portulaca grandiflora* and *Gaillardia grandiflora*	P	Plants of selected species were independently planted on ridge beds and watered with normal water for first 30 days	The textile effluent from common effluent treatment plant	1000 plants were nourished with normal water (control) and remaining 1000 plants with real textile wastewater for remaining 30 days of the experiments (test)	Reduction in dye by 59, 50, 46 and 73%, for each independent plant respectively within 30 days compared to dye accumulated in unplanted ridges	The mechanisms for their degradation or detoxification in plants are poorly understood	[90]
*Glandularia pulchella* (Sweet) Tronc. (Moss Verbena)	P	Plants of approximately the same growth stage, having equal number of shoots, and almost equivalent weight Three plants were dipped in 250 mL flasks containing solution of various dyes for 96 h	Decolorization of pollutant dyes	Three plants were dipped in each of the 250-mL SF containing 100 mL solution of the synthetic dye mixture for 96 h. Those are: reactive orange HE2R, reactive yellow MEG4, reactive yellow GR, blue 2GL, remazol red, green HE4B, brown 3REL, blue 2RNL, patent blue, and malachite green Cell free extract were used for enzyme assays	Plants of *G. pulchella* were exposed to solutions of ten different dyes and promoted the decolorization of all the dyes to varying extent	Biodegradation in living cells is realized by multiple enzymes (laccases and peroxidases mainly) to mineralize synthetic dyes	[88]
*Brumea malcolmii*	C F	Cell suspension cultures on modified Murashuge and Skoog’s medium pH 5.8 and 25 °C. The cultures were maintained at 100 rpm under 16:8 h light:darkness photoperiod during 10 days. Filtrates obtained were used as sources of extracellular enzymes for enzyme assays	Decolorization of pollutant dyes: brilliant blue R (BBR), malachite green, reactive red 2, direct red 5B and methyl orange	The respective dyes at 40 mg/L, 0.2 M sodium acetate buffer (pH 4.8) and 0.5 mL enzyme and 35 μM ABTS. The reaction mixture was incubated at 30 °C under static as well as shaking conditions at 100 rpm	Suspension cells and purified laccase showed the ability to decolorize different dyes completely. In the case of purified laccase, the addition of ABTS to BBR, increase decolorization and degradation	Whole cell cultures involved a asymmetric cleavage of BBR followed by a demethylation with laccase	[86]
*Alternanthera philoxeroides*	P	Plants were exposed to textile industry effluent in rhizofiltration reactor system for 6 days	Phytoremediation of sulfonated remazol red dye and textile effluents	Plants were put in contact with each effluent sample was monitored for a retention time of 6 days (144 h) and effluent samples were analyzed Cell free extract from roots, stem, leaves and plants as enzyme source	*A. philoxeroides* could completely decolorize remazol red dye and demonstrated potential in real textile industry effluent at laboratory and pilot scale	Unknown	[89]
Herbicide compounds							
Recombinant LAC1 from *Gossypium arboreum* in *Pichia pastoris* or transgenic *Arabidopsis thaliana*	*P*	Seeds of *A. thaliana* (ecotype Columbia) express LAC1 were germinated in a pot with either a syringic acid solution (2 mM) or a TCP solution (1 mM). Two weeks after germination, seedlings were sprayed with either the syringic acid solution every 3 days for 3 weeks, or with the TCP solution twice with a 5 days interval	Transformation of sinapic acid, to other phenolic compounds like 2,4,6-trichlorophenol (TCP)	LAC1 in presence of 60 nmol of ABTS/min/mg protein In transformed plants, expressing LAC1, 10–20 μM of TCP, syringic acid 2 mM or 0.5 mM of sinapic acid	Laccase activity was responsible for the conversion of sinapic acid into mono-lactone type dimer and the transformation of TCP	Conversion of sinapic acid into monolactone-type dimer	[84]
Recombinant laccases from *Oryza sativa* expressed in *Pichia pastoris*	CS	48 h cultures in YPD medium induced with 1% methanol at 30 °C	Modification and detoxification of herbicides atrazine (ATR) and isoproturon (IPU)	Transformants were add in YPD medium containing 1% methanol and 0.4 mg/L ATR or 2.0 mg/L IPU at 30 °C for 48 h. The ATR or IPU conversion rates were calculated	The heterologous expression of the two rice laccase genes in *P. pastoris* led to the cells resistant to ATR and IPU. This suggests that laccase could be involved in detoxification or degradation of ATR or IPU in plant	Mechanisms poorly understood	[91]

*P* whole plant, *R* plant roots, *CS* cell suspension, *C* crude culture supernatant, *F* free purified enzyme, *RGFL* rubin GF, *DCIP* 2,6-dichloroindophenol, *TCP* 2,4,6-trichlorophenol, *AzBTS-(NH4)2* 2,2′-azino-bis(3-ethylbenzothiazoline-6-sulfonic acid) diammonium salt, *ABTS* diammonium 2,2′-azino-bis(3-ethylbenzothiazoline-6-sulfonate), *BBR* brilliant blue R, *ATR* atrazine, *IPU* isoproturon, *YPD* yeast extract–peptone–dextrose

**Table 3:** Application of some interesting bacterial laccases that degrade different compounds and may be useful in water treatement

Laccase source	Applied enzyme form	Type of culture, ingredients	Application	Reaction parameters	Results obtained	Main putative mechanisms involved	References
Pharmaceutical compounds							
Recombinant laccase from *Yersinia enterocolitica* expressed in *E. coli*	CS	Expressed in *E. coli* with IPTG induction	Degradation of non-steroidal anti-inflammatory drugs	*E. coli* cells harboring laccase from *Y. enterocolitica* were treated with 0.1 mM Tween 80 and CuCl_2_ 0.2 mM in buffer pH 6, at 45 °C for 30 min. After that, diclofenac and aspirin were added at 5 mg/L incubated at 45 °C	After 24 h, both diclofenac and aspirin were fully degraded	In the case of the diclofenac, laccase oxidation by hydroxylations of 4′ or 5′ positions of the second benzene ring could be the modifications	[136]
*Streptomyces cyaneus*	C	Production of laccase was done in ISP9 mineral medium, with soy flour (10 g/L) as carbon source and a copper concentration of 1 mg/L CuSO_4_∙5 H_2_O. Cultures were incubated at 30 °C for 23 days. Cell-free culture supernatant was collected, filtered and stored as enzyme source	Degradation of non-steroidal anti-inflammatory drugs (diclofenac: DFC) and mefenamic acid: MFA)	The reactions were performed in citrate phosphate buffer (30–40 mM) at three different pH values (5, 6 and 7), with a pollutant concentration of 20 mg/L. 2000 U/L of crude enzyme preparation were used. The reactions were incubated in the dark at 25 °C for 12 days	The enzyme showed a high conversion rate under acidic conditions (pH 5, 6), with 50% of conversion after 2 days for DFC. With respect to MFA, the highest conversion was obtained in pH 6	Not discussed	[137]
Recombinant *Streptomyces ipomoea* SilA laccase expressed in *E. coli*	F	2 L of LB medium at 37 °C were inoculated with 40 mL of an exponential-growth-phase culture. When exponential growth had resumed, the temperature was reduced to 28 °C, and SilA expression was induced with 1 mM IPTG. Purified enzyme was used	Degradation of fluoroquinolone antibiotics (ciprofloxacin: CIP, and norfloxacin)	The reactions were carried out in 50 mM phosphate buffer pH 8 at 35 °C, using 0.4 U/mL of laccase and 50 μg/mL of each fluoroquinolone. Several mediators at concentrations of 0.1, 0.3 and 0.5 mM were tested	After 24 h and with 0.5 mM acetosyringone, higher than 90% percent conversions were obtained for both antibiotics, with a detoxification effectiveness of 70% for CIP and 90% for norfloxacin	Possible oxidation of piperazine substituents	[140]
*Streptomyces mutabilis* A17	F	The culture was done in a solid-state fermentation, using cotton seed cake (5 g/L) as substrate, supplemented with mineral salts and glucose 1% (w/v). The medium was inoculated with a spore suspension and incubated for 6 days at 35 °C. The laccase was extracted and purified	Degradation of sulfa antibiotics (sulfadiazine and sulfathiazole)	In a 100 mM citrate–phosphate buffer pH 6 were dissolved each sulfa drug, with a final concentration of 50 mg/L. To this solution were added the laccase (81.3 U/mg), and 1 mM HBT (mediator). The reaction was done at 50 °C for 60 min	Under the conditions previously described, 73 and 90% removal efficiencies were achieved to sulfadiazine and sulfamethoxazole solutions, respectively. Moreover, the reaction products showed less antibiotic effect in bacterial cultures	Not reported	[138]
Dye based pollutants							
Recombinant laccase from *Klebsiella pneumoniae* expressed in *E. coli*	F	*E. coli* cells were grown in LB medium at 37 °C until 0.6–0.8 DO. After that, were induced with IPTG for 20 h at 16 °C. Purified laccase	Decolorization of synthetic dyes	The reactions were done with 0.025, 0.05 and 0.1 U of the purified enzyme, in 50 mM citrate–phosphate buffer (pH 4.0 and 7.5) and 15 μL dye solution (100 mg/L) at 70 °C in 90 min	All the 10 dyes tested were efficiently oxidized under by the enzyme alone in both acidic and neutral conditions	Not reported	[99]
Recombinant *E. coli* K-12 CueO expressed in *Pichia pastoris*	F	48 h culture in BMGY at 28 °C. The induction was made with methanol 1% and 0.2 mM CuSO_4_ for 144 h, feeding methanol each 24 h. Purified laccase	Decolorization of synthetic dyes	The reactions were carried out at 55 °C in phosphates buffer 50 mM, pH 7.5, with a dye concentration of 80 mg/L and supplemented with 1 mM CuSO_4_ and 0.1 mM of acetosyringone as mediator. 1 μL of purified laccase was used	After 3 h of reaction, the laccase decolorized almost all the Congo red and malachite green tested. After 24 h, 90% of the remazol brilliant blue R were degraded	Not mentioned	[128]
Recombinant and mutant laccase WLF from *Bacillus pumilus* expressed in *E. coli*	F	Culture grown at 37 °C in LB medium until 0.5 DO. After that, were added IPTG (0.4 mM) and CuSO_4_ (0.25 mM) and maintained at 15 °C for 24 h. Purified laccase	Decolorization of synthetic dyes	Reaction mixture consisted in 0.25 mg of dye, 2 mg/L of purified laccase 1 mM de acetosyringone in 5 mL of 100 mM carbonate buffer pH 10, at 37 °C	Highest transformations of all the dyes tested. The efficiency with aromatic heterocyclic dyes was lower compared with azo, anthraquinonic and triphenylmethane dyes	Not reported	[125]
*Bacillus safensis* S31	SS	*B. saensis* cells were cultured on nutrient agar sporulation medium and incubated at 35 °C for 4 days. After that incubation time, spore suspension was prepared, used as a source of laccase	Decolorization of synthetic dyes (malachite green, toluidine blue and reactive black 5)	To 2 mL of 50 mM acetate buffer (for pH values of 3–6) or 50 mM Tris buffer (for pH values of 7 and 8) were added the spore laccase suspension (8 U/L) and dye (final concentration of 10 mg/L). The effect of ABTS (15 μM) as mediator was also studied. The reactions were carried out at 30 °C for 2 h	Almost all the oxidation conditions showed better results with ABTS. The highest decolorization values for malachite green and toluidine blue were achieved between 5 and 7 pH values, while with reactive black were between pH 3 and 5	Not mentioned	[134]
Recombinant *Thermus thermophilus* SG0.5JP17-16 expressed in *Pichia pastoris*	F	An inoculum of *Pichia pastoris* cells growth in BMGY medium were used to inoculate BMMY medium containing 0.1 mM CuSO_4_. The culture was cultivated at 30 °C for 7 days with daily addition of 1% methanol. The enzyme was purified from the supernatant	Decolorization of synthetic dyes (reactive black B, reactive black WNN, congo red and remazol brilliant blue R)	A reaction mixture of 50 mM phosphates buffer pH 7.5, 10 μM CuSO_4_, 50 mg/L dye and 40 U/L of purified laccase were heated at 70 °C for 24 h	After 24 h the decolorization efficiency for congo red, reactive black B and reactive black WNN was higher than 90%, while for remazol brilliant blue R was around 70%	Not mentioned	[126]
Recombinant *Streptomyces ipomoeae* SilA, expressed in *E. coli*	F	*E. coli* BL21 (DE3) transformed and containing the codifying gene of SilA 2 L of Luria–Bertani (LB)	Decolorization of synthetic dyes	Laccase SilA and three mediators (0.1 mM), acetosyringone (AS), syringaldehyde (SA) and methyl syringate (MeS), by 24 h at 35 °C pH 8, and different dyes (acid black 48: AB48, acid orange 63: AO63, reactive black 5: RB5, orange II: OII, tartrazine: TART, azure B: AB, indigo carmine: IC, cresol red: CR	Laccase and mediators such as AS and MeS enhanced the decolorization and detoxification of a variety of textile dyes, principally RB5, OII, and IC, diminishing the toxicity of acid orange 63, tartrazine	The oxidation of MeS (which has the weakest acceptor group at the para-position) gives an stable phenoxy radical	[139]
Plastic and polycyclic aromatic hydrocarbons (PAHs) compounds							
Recombinant *B. subtilis* CotA expressed in *E. coli*	F	*E. coli* cells harboring the plasmid with the CotA gen were grown at 37 °C in LB medium. When the culture reaches 0.6 DO, were added IPTG and CuSO_4_ to final concentrations of 0.1 and 0.25 mM, respectively. The incubation temperature was reduced to 25 °C for 6 h. After that, the culture agitation was stopped for 12 h. Purified laccase was used	Degradation of PAHs (anthracene, pyrene benzo[α]pyrene, phenanthrene, fluoranthene, etc.)	The reactions were carried out in 50 mM acetate buffer pH 4 with 10% acetonitrile, with PAHs concentrations from 0.1 to 1 mg/L and laccase concentration of 3 U/mL. The reactions were incubated for 24 h at 20, 40 and 60 °C	Just anthracene and benzo[α]pyrene were significantly oxidized (almost complete oxidations at 60 °C), the other ones had degradation values from 0 to 40% in all the conditions tested	Not reported	[124]
*Streptomyces cyaneus*	C	Production of laccase was done in ISP9 mineral medium, with soy flour (10 g/L) as carbon source and a copper concentration of 1 mg/L CuSO_4_∙5 H_2_O. Cultures were incubated at 30 °C for 23 days. Cell-free culture supernatant was collected, filtered and stored as enzyme source	Degradation of bisphenol A	The reactions were performed in citrate phosphate buffer (30–40 mM) at three different pH values (5, 6 and 7), with a pollutant concentration of 20 mg/L. 2000 U/L of crude enzyme preparation were used. The reactions were incubated in the dark at 25 °C for 12 days	Under all the conditions tested after 2 days there was full degradation, especially at pH 5 and 6	Not reported	[137]

*CS* cell suspension, *SS* spore suspension, *F* free purified enzyme, *C* crude enzyme extract, *IPTG* isopropyl β-d-1-thiogalactopyranoside, *DFC* diclofenac, *MFA* mefenamic acid, *AzBTS-(NH4)2* 2,2′-azino-bis(3-ethylbenzothiazoline-6-sulfonic acid) diammonium salt, *ABTS* diammonium 2,2′-azino-bis(3-ethylbenzothiazoline-6-sulfonate), *PAHs* polycyclic aromatic hydrocarbons, *BMGY* buffered glycerol-complex medium, *CIP* ciprofloxacin, buffered methanol-complex medium

## Concluding remarks

There is currently an urgent need to reverse the pollution of global water bodies caused by humans. Despite the diversity of pollutants discharged into waterbodies, such as plastics, herbicides, fertilizers, synthetic dyes, phthalates, and pharmaceuticals, laccases appear to be an efficient biocatalytic tool with the ability to oxidize these molecules, yielding less-toxic and harmful inactive compounds. Some limiting factors, such as the complex composition of contaminated water (high concentrations of salts and/or high pH values), can be overcome by engineering laccases using modern techniques like in vitro evolution and site-directed mutagenesis, enhanced by theoretical tools such as molecular modeling, and dynamic simulations, among others. Not only engineering well-known enzymes, but also searching for new enzymes, with new properties, can provide guidance on the use or design of new structural and biochemical mechanisms of action of the biocatalysts, improving their application in the bioremediation and biotransformation of contaminated waterbodies.

Existing literature suggests that the degradation of emerging pollutants can be achieved (including that of hard-to-degrade molecules, such as nonsteroidal anti-inflammatory drugs or PAHs), but the practical implementation of the degrading bioprocesses is always cost-dependent. Conventional approaches to immobilize laccases, as well as those to extract and purify them, are time-consuming and can be expensive. However, the high-yield production of recombinant laccases, with elevated activity and stability and a decrease in the number of steps for enzyme pre-purification would be advantageous in terms of cost-effectiveness.

## Data Availability

All information is included in this published article.
